# Secondary Metabolites with Agricultural Antagonistic Potential from *Aspergillus* sp. ITBBc1, a Coral-Associated Marine Fungus

**DOI:** 10.3390/md22060270

**Published:** 2024-06-11

**Authors:** Ailiman Abulaizi, Rong Wang, Zijun Xiong, Shiqing Zhang, Yuanchao Li, Huiming Ge, Zhikai Guo

**Affiliations:** 1 State Key Laboratory of Pharmaceutical Biotechnology, Institute of Functional Biomolecules, Chemistry and Biomedicine Innovation Center (ChemBIC), School of Life Sciences, Nanjing University, Nanjing 210023, China; helime5052@163.com; 2 Hainan Key Laboratory of Tropical Microbe Resources, Institute of Tropical Bioscience and Biotechnology, Chinese Academy of Tropical Agricultural Sciences & Key Laboratory for Biology and Genetic Resources of Tropical Crops of Hainan Province, Hainan Institute for Tropical Agricultural Resources, Haikou 571101, China; wangrong1982@gmail.com (R.W.); xiongzijun@itbb.org.cn (Z.X.); zhangshiqing@itbb.org.cn (S.Z.); 3 Hainan Provincial Key Laboratory of Tropical Maricultural Technologies, Hainan Academy of Ocean and Fisheries Sciences, Haikou 571126, China

**Keywords:** *Aspergillus* sp., marine fungi, secondary metabolites, phytotoxic activity

## Abstract

A marine-derived fungal strain, *Aspergillus* sp. ITBBc1, was isolated from coral collected from the South China Sea in Hainan province. Intensive chemical investigation of the fermentation extract of this strain afforded four new secondary metabolites (**1**–**4**), named megastigmanones A–C and prenylterphenyllin H, along with four known compounds (**5**–**8**). Their structures were elucidated by extensive spectroscopic analysis including one-and two-dimensional (1D and 2D) NMR spectroscopy and high-resolution electrospray ionization mass spectrometry (HR-ESI-MS). The modified Mosher’s method was undertaken to determine the absolute configurations of new compounds. The phytotoxic activity test showed that compounds **6**–**8** exhibited significant antagonistic activity against the germination of *Triticum aestivum* L. and *Oryza sativa* L. seeds with a dose-dependent relationship.

## 1. Introduction

Fungi are ubiquitous in nature, with a wide variety and large quantities. Many fungi are beneficial to the environment, humans and animals, while some are harmful due to the production of toxic metabolites (mycotoxins) [1]. More than 300 mycotoxins have been identified so far [2], and most of them are produced by well-known genera including *Aspergillus*, *Fusarium* and *Penicillium* [3,4,5,6,7]. Mycotoxins not only carry the potential to cause contaminations and result in serious annual yield and quality losses but also pose life-threatening risks to human and animal health [8]. Therefore, they have become a major issue in the sciences [9,10]. With synthetic agrochemicals becoming increasingly controversial due to their environmental and toxicological problems [11], mycotoxins have drawn great attention because of their potential as natural herbicides [12,13,14]. Modern agriculture heavily relies on natural herbicides for weed control due to their high efficiency and low cost [15]. Thus, herbicidal mycotoxins are considered environmentally friendly and sustainable alternative agents for controlling weeds. However, little is known about the occurrence and mechanisms of biosynthesis under various environmental conditions [16]. Therefore, excavating these mycotoxins has a vital impact on exploring the great potential of new natural herbicides.

With the expansion of research scope, more and more fungal mycotoxins with herbicidal potential have been discovered from marine fungi [17,18]. Many fungal mycotoxins can also be used as lead compounds, based on their structure, to obtain more active toxic compounds through chemical synthesis. The synthesis of Cinnacidin analogues is a typical case, and the synthesized analogues have better phytotoxic activity against weeds, demonstrating their potential application value as herbicides [19].

**Figure 1:**
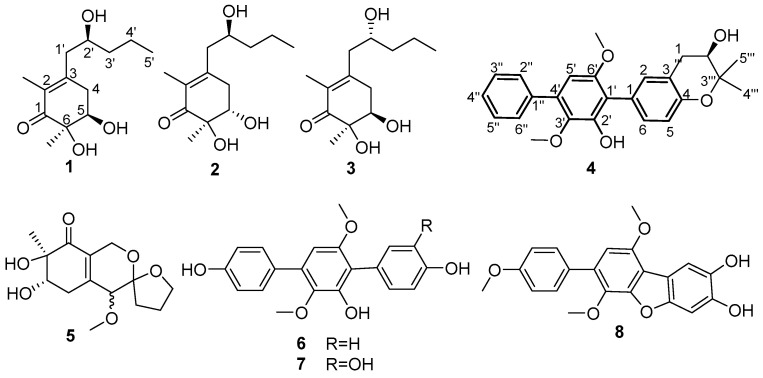
Chemical structures of compounds **1**–**8**.

As part of our continuing exploration of structurally novel and biologically interesting secondary metabolites from marine-derived microorganisms [20,21,22], a coral-associated fungus, *Aspergillus* sp. ITBBc1, has drawn our attention. Furthermore, intensive chemical investigation of this fungus led to the identification of four new compounds (**1**–**4**) and four known compounds (**5**–**8**) (Figure 1). Herein, we report the isolation, structural elucidation, and biological activity evaluation of these compounds.

## 2. Results

The EtOAc extract of the fungus *Aspergillus* sp. ITBBc1 was subjected to repeated column chromatography (CC) (silica gel, Sephadex LH-20) and semi-preparative HPLC to yield four new compounds (**1**–**4**) along with four known analogues (**5**–**8**). The known compounds were rapidly characterized as peniazaphilin A (**5**) [23], terphenyllin (**6**) [24], 3-hydroxyterphenyllin (**7**) [25], and candidusin C (**8**) [26] (Figure 1), respectively, via comparison of their experimental spectroscopic data with those reported in the literature.

### 2.1. Structural Elucidation

**Figure 2:**
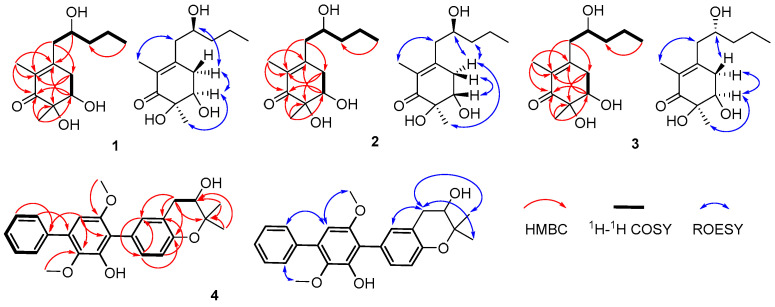
Key HMBC, ^1^H-^1^H COSY and ROESY correlations of new compounds **1**–**4**.

**Figure 3:**
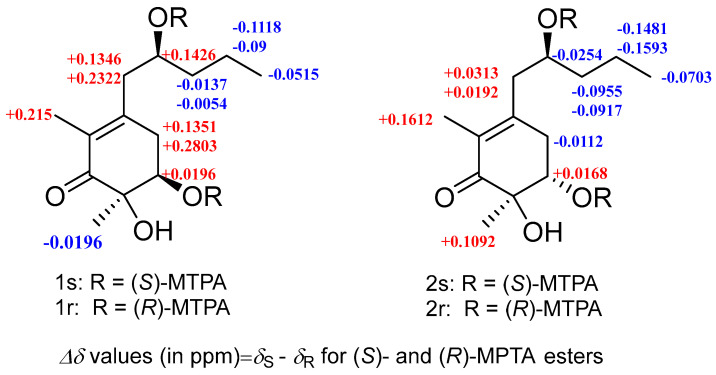
Δ*δ^RS^* values for the MTPA derivatives of compounds **1** and **2** in pyridine-*d*_5_.

Megastigmanone A (**1**) was obtained as colorless oil. Its molecular formula, C_13_H_22_O_4_, was determined based on the HR-ESI-MS ion at *m*/*z* 265.1410 [M + Na]^+^ (calcd, C_13_H_22_NaO_4_, 265.1410), indicating three degrees of unsaturation. The ^1^H NMR spectrum (Figure S1) revealed the existence of three methyls at *δ*_H_ 1.79 (3H, s, CH_3_-2), 1.23 (3H, s, CH_3_-6), and 0.91 (3H, t, *J* = 7.1Hz, H-5′), two oxygenated protons at *δ*_H_ 3.90 (1H, m, H-2′) and 3.98 (1H, dd, *J* = 3.7, 2.9 Hz, H-5), eight aliphatic protons and three hydroxyl groups (Table 1). The ^13^C and DEPT135 NMR data showed that there were 13 carbon resonances, which were assigned to one carbonyl at *δ*_C_ 202.2 (C-1), three nonprotonated carbons at *δ*_C_ 152.7 (C-3), 128.8 (C-2), and 76.4 (C-6), two methines at *δ*_C_ 74.2 (C-5) and 70.0 (C-2′), four methylenes at *δ*_C_ 43.7 (C-1′), 41.0 (C-3′), 37.1 (C-4) and 19.6 (C-4′), and three methyls at *δ*_C_ 23.3 (CH_3_-6), 14.3 (C-5′) and 11.6 (CH_3_-2) (Table 1). The planar structure of compound **1** was proposed by HMBC (Figure S5) and ^1^H-^1^H COSY spectra (Figure S6). Analysis of the ^1^H-^1^H COSY spectrum revealed the existence of two separate spin systems: H_2_-4-H-5 and H_2_-1′-H_3_-5′ (Figure 2). HMBC correlations from H-4 to C-2, C-3 and C-6, from H-5 to C-1, C-3, C-6 and CH_3_-5′, from H-1′ to C-2, C-3, C-4 and C-3′, from H-2′ to C-3, from H-4′ to C-2′, and from H-5′ to C-3′ were observed. The HMBC correlation from CH_3_-2 to C-1, C-2 and C-3 suggested the methyl group at C-2, and that from CH_3_-6 to C-1, C-5 and C-6 suggested the methyl group at C-6. The ROESY correlations of H-5/CH_3_-6, H-5/H_2_-4,H-4/H-2′ and CH_3_-2/H_2_-1′ (Figure 2) could further support the relative configuration. The whole interpretation of 1D and 2D NMR data allowed the confirmation of the planar structure of compound **1**, as shown in Figure 1. In order to further determine the absolute configuration of compound **1**, the modified Mosher’s method was used to determine the absolute configurations of secondary alcohols at C-5 and C-2′. Compound **1** was reacted with (*R*)/(*S*)-MTPA-Cl to obtain (*S*) and (*R*)-Mosher esters. The difference in chemical shifts of the Mosher esters of compound **1** indicate that the stereocenters at C-5 and C-2′ were *R* and *S* (Figure 3; Figures S8–S11), respectively. Therefore, the complete structure of compound **1** was established as (5*R*, 6*R*)-5,6-dihydroxy-3-((*S*)-2′-hydroxypentyl)-2, 6-dimethylcyclohex-2- en-1-one.

Megastigmanone B (**2**) was isolated as colorless oil. Its molecular formula, C_13_H_22_O_4_, was determined based on the HR-ESI-MS ion at *m*/*z* 265.1447 [M + Na]^+^ (calcd, C_13_H_22_NaO_4_, 265.1410), indicating three degrees of unsaturation. Comparison of its 1D and 2D NMR data with those for compound **1** revealed that compound **2** shared the same skeleton with compound **1**, except for the different configurations of H-5, revealed by the ROESY correlation between CH_3_-6 and H-5 in **2**. The absolute configuration of C-5 and C-2′ in compound **2** was also determined by the modified Mosher’s method and has been determined to be (5*S*, 6*R*)-5, 6-dihydroxy-3-((*S*)-2′-hydroxypentyl)-2, 6-dimethylcyclohex-2-en-1-one according to the Δ*δ_RS_* value analysis of the ^1^H NMR data of (*S*) and (*R*)-MTPA esters of compound **2** (Figure 3; Figures S20–S23).

Megastigmanone C (**3**) was also obtained as colorless oil. Its molecular formula, C_13_H_22_O_4_, was determined based on the HR-ESI-MS ion at *m*/*z* 243.1595 [M + H]^+^ (calcd, C_13_H_23_O_4_, 243.1591), indicating three degrees of unsaturation. The 1D and 2D NMR data for compound **3** (Figures S26–S31) were very similar to those of compound **1**, allowing the determination of the same planar structure as compounds **1** and **2**, except for the different configuration of H-2′ through comparing the carbon values (C-5s in compounds **1** and **3** were *δ*_C_ 74.2, while the C-2′ in **3** was downshifted to *δ*_C_ 70.4) and ROESY data. Efforts were undertaken to establish the absolute configuration of C-2′ through the application of the modified Mosher’s method. However, these attempts were not successful. According to the ROESY experiment, the whole structure for **3** was tentatively determined to be (5*R*, 6*R*)-5,6-dihydroxy-3-((*R*)-2′-hydroxypentyl)-2, 6-dimethylcyclohex-2-en-1-one.

Prenylterphenyllin H (**4**) was isolated as a yellow amorphous solid. Its molecular formula, C_25_H_26_O_5_, was determined based on the HR-ESI-MS ion at *m*/*z* 429.1673 [M + Na]^+^ (calcd, C_25_H_26_NaO_5_, 429.1672), indicating 13 degrees of unsaturation. The ^1^H NMR spectrum (Figure S39) revealed the existence of two methyls at *δ*_H_ 1.38 (3H, s, C-4‴) and 1.28 (3H, s, C-5‴), two oxygenated methyls at *δ*_H_ 3.74 (3H, s, CH_3_-6′) and 3.38 (3H, s, CH_3_-3′), and nine olefinic protons at *δ*_H_ 7.67 (2H, d, *J* = 7.4 Hz, H-2″,6″), 7.46 (2H, t, *J* = 7.4 Hz, H-3″,5″), 7.38 (1H, t, *J* = 7.4 Hz, H-4″), 7.14 (1H, dd, *J* = 8.3, 2.0 Hz, H-6), 7.11 (1H, d, *J* = 2.0 Hz, H-2), 6.74 (1H, d, *J* = 8.3 Hz, H-5) and 6.53 (1H, s, H-5′) (Table 2). The ^13^C and DEPT135 NMR data showed that there were 25 carbon resonances, which were assigned to ten nonprotonated carbons at *δ*_C_ 154.6 (C-6′), 152.9 (C-4), 149.2 (C-2′), 140.3 (C-3′), 139.5 (C-1″), 133.6 (C-4′), 126.4 (C-1), 120.2 (C-3), 118.4 (C-1′) and 77.8 (C-3‴), ten methines at *δ*_C_ 133.0 (C-2), 131.0 (C-6), 129.7 (C-2″, 6″), 129.2 (C-3″,5″), 128.1 (C-4″), 116.7 (C-5), 104.3 (C-5′), and 70.1 (C-2‴), one methylene at *δ*_C_ 32.3 (C-1‴), two methyl carbons at *δ*_C_ 26.7 (C-5‴) and 20.7 (C-4‴), and two methoxyls at *δ*_C_ 60.9 (OCH_3_-3′) and 20.7 (OCH_3_-6′) (Table 2). These NMR data were very similar with those of the previously reported sanshamycin C [20], except for the presence of a segment of -CH_2_-CHOH- through detailed analysis of the HMBC (Figure S43) and ^1^H-^1^H COSY spectra (Figure S44) of compound **4**. Thus, the planar structure of compound **4** was proposed to be sansamycin C’s derivative. Analysis of the ^1^H-^1^H COSY spectrum revealed the existence of three separate spin systems: H-2″-H-6″, H-5-H-6 and H_2_-1‴-H-2‴ (Figure 2). The HMBC correlations from OCH_3_-3′ to C-3′, from OCH_3_-6′ to C-6′, and from CH_3_-4‴ to C-3‴, C-5‴, C-2‴, CH_3_-5‴ to C-3‴, C-4‴, C-2‴ and ROESY correlations from OCH_3_-3′ to H-6″, OCH_3_-6′ to H-5′, CH_3_-4‴and CH_3_-5‴ to H-1‴ revealed the position of four methyl groups. Overall analysis of the 1D and 2D NMR data permitted the structural assignment for compound **4**, as shown in Figure 1. Attempts were also made to determine the absolute configuration of C-2‴ using the modified Mosher’s method, but with no success. So, we tried to compare the optical rotation of compound **4** with those of compounds bearing similar chiral moieties in the literature, such as clausenanisine B [27] and (−) microphyline Q [28], indicating *R* for C-2‴.

### 2.2. Antagonistic Evaluation

**Figure 4:**
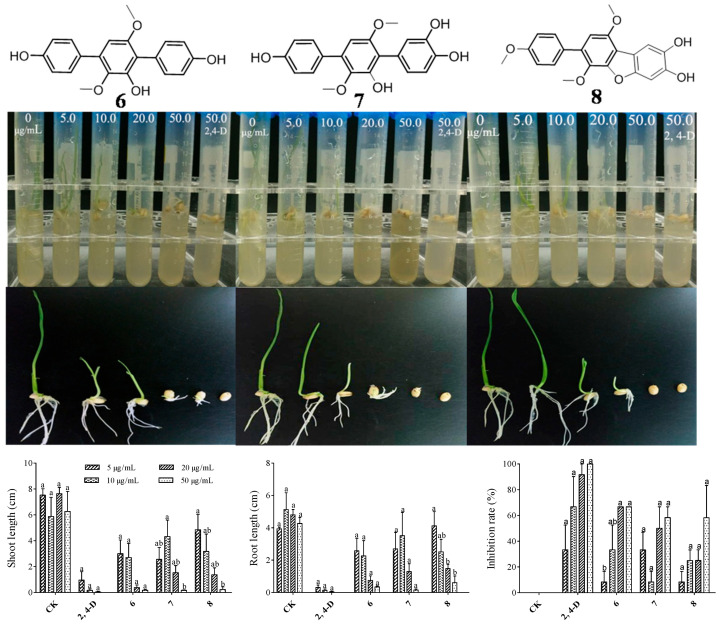
Phytotoxic activity of compounds **6**–**8** against *Triticum aestivum* L. Different letters show statistical significance.

**Figure 5:**
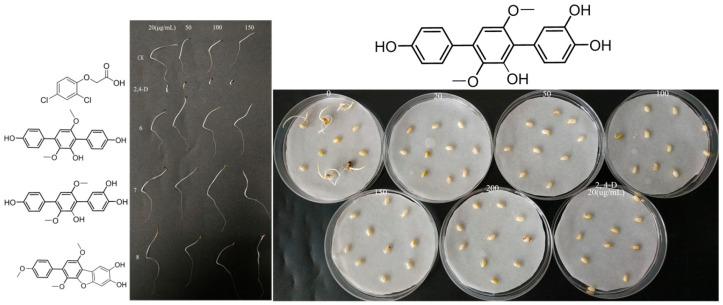
Phytotoxic activity of compounds **6**–**8** against *Amaranthus retroflexus* L. (**left**); phytotoxic activity of compound 7 against *Oryza sativa* L. (**right**).

The phytotoxic activity of the isolated compounds was tested using *Triticum aestivum* L. (monocots), *Oryza sativa* L. (monocots) and *Amaranthus retroflexus* L. (dicotyledon) seeds. As a result, *p*-terphenyl derivatives of compounds **6**–**8** exhibited significant antagonistic activities against the germination of *Triticum aestivum* L. and *Oryza sativa* L. seeds as well as the root and shoot lengths of seedlings with a dose-dependent relationship (Figure 4 and Figure 5). Compound **7** exhibited almost identical phytotoxic activity, indicating that the hydroxyl group at position C-5 has no impact on their phytotoxicity. However, they have no significant effects against *Amaranthus retroflexus* L. 

Combining the germination morphology and inhibition rate, as shown in Figure 4, it was found that there was a significant difference between *Triticum aestivum* L. seeds treated with 20 μg/L of compounds **6**–**8** and below 20 μg/L and the control. Shoot growth inhibition is more obvious than that of roots. The germination rate of *Oryza sativa* L. seeds treated with higher concentrations (50–200 μg/L) was significantly lower than the control or even no germination, and the development of roots and shoots was significantly inhibited compared to the control. The germination rate as well as the root and shoot lengths of seedlings of *Amaranthus retroflexus* L. seeds treated with higher concentrations (20–150 μg/L) were not affected. By comparing the inhibitory effects of compound **7** on *Triticum aestivum* L. and *Oryza sativa* L. seeds, it was found that *Oryza sativa* L. seeds are more sensitive to the treatment and can be effectively inhibited by compound **7** at a low concentration (20 μg/L). To sum up, the results indicated that the minimum inhibitory concentration for three germination inhibitors with significant inhibitory effects against *Triticum aestivum* L. is 20 μg/L. However, they have no significant inhibitory effects against *Amaranthus retroflexus* L., even at concentrations up to 150 μg/L. Therefore, it can be concluded that these compounds have significant selectivity for monocotyledonous and dicotyledonous plants seeds, and it is implied that they are more possibly only phytotoxic against monocots.

## 3. Materials and Methods

### 3.1. General Experimental Procedures

NMR spectra were recorded using a Bruker AVANCEIII-500 NMR spectrometer at 500 MHz for ^1^H and 125 MHz for ^13^C nuclei (Bruker Corporation, Karlsruhe, Germany). HRESIMS data were determined using an Agilent 6530 TOF LC-MS mass spectrometer (Agilent, Santa Clara, CA, USA) and Bruker compact mass spectrometer (Bruker, Bremen, Germany). Optical rotation values were recorded using an Anton Paar MCP5100 (Anton Paar, Germany). IR data were measured on a Nicolet 380 Infrared Spectrometer (Thermo Electron Corporation, Madison, WI, USA). UV and ECD data were collected on an MOS-500 Bio-Logic circular dichroic spectrometer (Bio-Logic, Seyssinet-Pariset, France). The semi-preparative HPLC was conducted on a Waters 1525 HPLC equipped with a XBridge C_18_ column (5 μm, 250.0 mm × 10.0 mm; Waters Corporation, Milford, MA, USA). Thin-layer chromatography (TLC) was performed on pre-coated glass plates (silica gel GF254, Qingdao Marine Chemical Inc., Qingdao, China). Column chromatography (CC) was performed on silica gel (45–75 μm; Qingdao Marine Chemical Inc., Qingdao, China), ODS (40–60 μm; Osaka Soda Co., Ltd., Hyogo, Japan) and Sephadex LH-20 (Cytiva, Uppsala, Sweden).

### 3.2. Fungal Material and Fermentation

The symbiotic epiphytic fungal strain of *Aspergillus* sp. ITBBc1 was previously isolated from coral collected from the South China Sea in Hainan and identified as *Aspergillus* sp. by internal transcribed spacer (ITS) sequencing (GenBank accession number OP614945) [20]. The strain was cultivated on ME medium (malt extract 10.0 g/L, sucrose 10.0 g/L, tryptone 1.0 g/L) cultured on a rotary shaker (180 rpm) at 28 °C for 3 days to afford a seed culture, which was then inoculated into rice solid media (rice 90.0 g and 0.1 L water) in 1L-Erlenmeyer flasks and fermented at 28 °C for 45 d under static conditions.

### 3.3. Extraction and Isolation 

The fungal culture was exhaustively extracted using ethyl acetate (EA) to obtain a crude extract, which was fractionated via silica gel CC with the eluting gradient of petroleum ether (PE)/EA(100:0, 50:1, 25:1, 10:1, 5:1, 2:1, 1:1, 0:1) to yield eight fractions (Fractions (Frs.) A–H). Fraction F (Fr. F) was purified by Sephadex LH-20 (MeOH) and then further purified by using semipreparative HPLC to obtain compound **4** (1.8 mg, *t*_R_ 15.8 min) (80% MeOH–H_2_O). Fraction G (Fr. G) was purified via CC over RP-C18 eluting with a MeOH–H_2_O gradient (from 4:6 to 1:0) to obtain six subfractions (Fr. G-1 to G-6). G-4 was purified by Sephadex LH-20 (MeOH) and then further purified by using semipreparative HPLC (40% MeOH–H_2_O) to obtain compounds **1** (7.8 mg, *t*_R_ 15.8 min), **2** (5.7 mg, *t*_R_ 18.1 min), and **3** (3.3 mg, *t*_R_ 23.8 min).

#### 3.3.1. Megastigmanone A (**1**)

Colorless oil; [α]${\;\;}_{D}^{25}$ −9.0 (*c* 0.10, MeOH); UV (MeOH) *λ*_max_ (log ε) 257 (2.32), 260 (2.29) nm (Figure S13); CD (c 0.05, MeOH) *λ*_max_ (∆ε): 233 (−3.61), 266 (+0.27), 315 (−0.51) nm (Figure S49). IR (KBr) *ν*_max_ 3396, 2931, 1669, 1089 cm^−1^ (Figure S14); ^1^H and ^13^C NMR data, see Table 1; HR-ESI-MS *m*/*z*: 265.1441 [M + Na]^+^ (calcd for C_13_H_22_NaO_4_, 265.1410) (Figure S12).

#### 3.3.2. Megastigmanone B (**2**)

Colorless oil; [α]${\;\;}_{D}^{25}$ +42.0(*c* 0.10, MeOH); UV (MeOH) *λ*_max_ (log ε) 251 (2.36), 266 (2.01) nm (Figure S27); CD (*c* 0.05, MeOH) *λ*_max_ (∆ε): 214(−7.04), 251 (+6.36), 319 (−0.32) nm (Figure S49). IR (KBr) *ν*_max_ 2958, 2930, 1664, 1095 cm^−1^ (Figure S28); ^1^H and ^13^C NMR data, see Table 1; HR-ESI-MS *m*/*z*: 265.1447 [M + Na]^+^ (calcd, C_13_H_22_NaO_4_, 265.1410) (Figure S26).

#### 3.3.3. Megastigmanone C (**3**)

Colorless oil; [α]${\;\;}_{D}^{25}$ −24.0 (*c* 0.10, MeOH); UV (MeOH) *λ*_max_ (log ε) 251 (2.24), 265 (1.97) nm (Figure S37); CD (*c* 0.05, MeOH) *λ*_max_ (∆ε): 242 (−4.90), 285 (+0.17) nm (Figure S49). IR (KBr) *ν*_max_ 3484, 2922, 1725, 1660, 1385 cm^−1^ (Figure S38); ^1^H and ^13^C NMR data, see Table 1; HR-ESI-MS *m*/*z*: 243.1595 [M + H]^+^ (calcd for C_13_H_23_O_4_, 243.1591) (Figure S36).

#### 3.3.4. Prenylterphenyllin H (**4**)

Yellow amorphous solid; [α]${\;\;}_{D}^{25}$ −14.0 (*c* 0.10, MeOH); UV (MeOH) *λ*_max_ (log ε) 222 (2.56), 249 (2.32), 273 (2.39) (Figure S47); CD (*c* 0.05, MeOH) *λ*_max_ (∆ε): 220 (−1.01), 229 (−2.20), 249 (−1.62) nm; IR (KBr) *ν*_max_ 3420, 2924, 1617, 1066 cm^−1^ (Figure S48); ^1^H and ^13^C NMR data, see Table 2; HR-ESI-MS *m*/*z*: 429.1673 [M + Na]^+^ (calcd, C_25_H_26_NaO_5_, 429.1672) (Figure S46).

### 3.4. Modified Mosher’s Reaction

The (*S*)-MTPA and (*R*)-MTPA esters for compounds **1**–**4** were prepared using modified Mosher’s method as previously reported [29]. Samples of compound **1** (1.0 mg, 0.0041 mmol) in pyridine-*d*_5_ (0.5 mL) and (*R*)-MTPACl (7.8 μL, 0.0410 mmol), compound **2** (1.0 mg, 0.0041 mmol) in pyridine-*d*_5_ (0.5 mL) and (*R*)-MTPACl (7.8 μL, 0.0410 mmol), compound **3** (1.0 mg, 0.0044 mmol) in pyridine-*d*_5_ (0.5 mL) and (*R*)-MTPACl (8.2 μL, 0.0440 mmol), and compound **4** (1.0 mg, 0.0041 mmol) in pyridine-*d*_5_ (0.5 mL) and (*R*)-MTPACl (4.6 μL, 0.0410 mmol) were reacted in NMR tubes at room temperature for 24 h, respectively. (*R*)-MTPA esters were also prepared in a similar way using (*S*)-MTPACl.

### 3.5. Antagonistic Bioassay 

The *Triticum aestivum* L. (monocots), *Oryza sativa* L. (monocots) and *Amaranthus retroflexus* L. (dicotyledon) seeds were disinfected with 8% sodium hypochlorite solution for 15 min and then washed three times with sterilized water. Then, *Triticum aestivum* L. seeds were placed in a 15 mL tube containing 5 mL of 1/2 MS medium with four concentrations (0, 5, 10, 20, and 50 μg/mL). *Oryza sativa* L. and *A. retroflexus* seeds were evenly placed on sterilized filter paper in separate 120 mm Petri dishes containing 2 mL of compound **7** with four concentrations (0, 20, 50, 100, 150, and 200 μg/mL). Seeds cultivated at a 25 ℃ growth chamber for upright growth for 5 d (16 h/8 h light/dark cycle). Then, the shoot length and root length of each treatment were measured, and the germination inhibition rates were calculated. The commonly used synthetic herbicide 2, 4-dichlorophenoxyacetic acid (2, 4-D) was used as a positive control. The inhibition percentage was calculated using the formula as follows:

inhibition percentage (%) = (L_control_ − L_treatment_)/L_control_ × 100

## 4. Conclusions

In summary, this study focused on the isolation and structural elucidation of four new compounds and four known compounds from the marine fungus *Aspergillus* sp. ITBBc1. The identification of these compounds was accomplished through the application of diverse spectroscopic techniques and comparison with published data. Compounds **1**–**3** have a similar structure to compounds reported by González Coloma et al., obtained from *Stemphylium solani* [30], which have biocidal activity simultaneously against more than one category of harmful organisms affecting plants. Compound **4** belongs to *p*-terphenyl, possessing the same skeleton with prenylterphenyllin G [31] and modified by hydroxy group at C-2‴. Compound **5** is an anazaphilone derivative that was first reported by Zhang et al. [23]. Compounds **6** and **7** are *p*-terphenyl derivatives and have the ability to inhibit wheat coleoptile growth significantly according to the previous reports [32]. Compound **7** has also been reported as an important anti-proliferative and pro-apoptotic agent for ovarian cancer [33]. Compound **8** is a terphenyllin derivative. Our results showed that compounds **6**–**8** exhibited remarkable antagonistic activities against the germination of *Triticum aestivum* L. and *Oryza sativa* L. seeds, but did not show effects against and *Amaranthus retroflexus* L. Therefore, it can be roughly concluded that compounds **6**–**8** are only phytotoxic against monocots. These bioactive compounds can be considered for weed control as herbicides in the field of dicotyledonous crops as they are only toxic to monocots, so they can be mixed with fertilizer to inhibit the germination of monocotyledonous weeds in the soil. Taken together, the unique structures of new compounds and the biologically active compound could provide an interesting foundation for further investigations. 

## Figures and Tables

**Table 1:** ^1^H (500 MHz) and ^13^C (125 MHz) NMR data for compounds **1**–**3** in acetone-*d*_6_.

Position	1		2		3	
	*δ*_C_	*δ*_H_ (*J* in Hz)	*δ*_C_	*δ*_H_ (*J* in Hz)	*δ*_C_	*δ*_H_ (*J* in Hz)
1	202.2, C		202.9, C		202.2, C	
2	128.8, C		129.4, C		128.6, C	
2-CH_3_	11.6, CH_3_	1.79 (s)	11.7, CH_3_	1.78 (s)	11.5, CH_3_	1.79 (s)
3	152.7, C		155.8, C		153.1, C	
4	37.1, CH_2_	2.86 (m); 2.63 (m)	38.6, CH_2_	2.75 (ddd, 18.4, 5.7, 1.1); 2.45 (m)	38.2, CH_2_	2.90 (d, 19.0); 2.58 (d, 19.0)
5	74.2, CH	3.97 (dd, 3.7, 2.9)	72.9, CH	3.85 (dd, 10.2, 5.7)	74.2, CH	3.96 (dd, 3.7, 2.9)
5-OH		3.70 (br s)		3.89 (br s)		3.74(br s)
6	76.4, C		77.8, C		76.4, C	
6-CH_3_	23.3, CH_3_	1.23 (s)	18.2, CH_3_	1.15 (s)	23.4, CH_3_	1.22 (s)
6-OH		4.17 (br s)		4.19 (br s)		4.13 (br s)
1′	43.7, CH_2_	2.51 (dd, 13.3, 8.7) 2.35 (dd, 13.3, 4.2)	43.6, CH_2_	2.45 (m)	43.9, CH_2_	2.47 (dd, 13.1, 4.7) 2.34 (dd, 13.1, 8.4)
2′	70.0, CH	3.89 (m)	70.1, CH	3.89 (m)	70.4, CH	3.85 (m)
2′-OH		3.70 (br s)		3.04 (br s)		3.67 (br s)
3′	41.0, CH_2_	1.47 (m)	41.3, CH_2_	1.48 (m)	41.1, CH_2_	1.48 (m)
4′	19.6, CH_2_	1.51 (m); 1.40 (m)	19.6, CH_2_	1.50 (m); 1.40 (m)	19.6, CH_2_	1.51 (m); 1.41 (m)
5′	14.3, CH_3_	0.91 (t, 7.1)	14.3, CH_3_	0.92 (t, 7.1)	14.4, CH_3_	0.91 (t, 7.1)

**Table 2:** ^1^H (500 MHz) and ^13^C (125 MHz) NMR data for compound **4** in acetone-*d*_6_.

Position	*δ*_C_	*δ*_H_ (*J* in Hz)	Position	*δ*_C_	*δ*_H_ (*J* in Hz)
1	126.4, C		1″	139.5, C	
2	133.0, CH	7.11 (d,2.0)	2″	129.7, CH	7.67 (d, 7.4)
3	120.2, C		3″	129.2, CH	7.46 (t, 7.4)
4	152.9, C		4″	128.1, CH	7.38 (t,7.4)
5	116.7, CH	6.74 (d, 8.3)	5″	129.2, CH	7.46 (t, 7.4)
6	131.0, CH	7.14 (dd, 8.3, 2.0)	6″	129.7, CH	7.67 (d, 7.4)
1′	118.4, C		1‴	32.3, CH_2_	3.01 (dd, 16.4, 5.4); 2.76 (dd, 16.4, 8.2)
2′	149.2, C		2‴	70.1, CH	3.82 (dd, 7.5, 5.3)
3′	140.3, C		3‴	77.8, C	
3′-OCH_3_	60.9, CH_3_	3.38 (s)	4‴	20.7, CH_3_	1.28 (s)
4′	133.6, C		5‴	26.3, CH_3_	1.38 (s)
5′	104.3, CH	6.53 (s)			
6′	154.6, C				
6′-OCH_3_	56.1, CH_3_	3.74 (s)			

## Data Availability

Data are contained within the article or Supplementary Materials; further inquiries can be directed to the corresponding author.

## Supplementary Materials

The following supporting information can be downloaded at https://www.mdpi.com/article/10.3390/md22060270/s1: Figures S1–S49: HR-ESI-MS, ^1^H NMR, ^13^C NMR, DEPT 135, HSQC, HMBC, ^1^H-^1^H COSY, ROESY, UV, IR spectra of compounds **1**–**4** and ECD spectra of compounds **1**–**3**.
