# Supplementary material for: Secondary Metabolites with Agricultural Antagonistic Potential from Aspergillus sp. ITBBc1, a Coral-Associated Marine Fungus
Source: Mar Drugs. 2024 Jun 11;22(6):270. doi: 10.3390/md22060270 (PMC11205182; doi:10.3390/md22060270)

Secondary Metabolites with Agricultural Antagonistic Potential from *Aspergillus* sp. ITBBc1, a Coral-Associated Marine Fungus

Ailiman Abulaizi [1], Rong Wang[3], Zijun Xiong [2], Shiqing Zhang[2], Yuanchao Li[3,*], Hui Ming Ge [1,*], Zhikai Guo [2,*]

[1] State Key Laboratory of Pharmaceutical Biotechnology, Institute of Functional Biomolecules, Chemistry and Biomedicine Innovation Center (ChemBIC), School of Life Sciences, Nanjing University, Nanjing 210023, China; helime5052@163.com (A.A.)

[2] Hainan Key Laboratory of Tropical Microbe Resources, Institute of Tropical Bioscience and Biotechnology, Chinese Academy of Tropical Agricultural Sciences & Key Laboratory for Biology and Genetic Resources of Tropical Crops of Hainan Province, Hainan Institute for Tropical Agricultural Resources, Haikou 571101, China; xiongzijun@itbb.org.cn (Z.X.); zhangshiqing@itbb.org.cn (S.Z.)

[3] Hainan Provincial Key Laboratory of Tropical Maricultural Technologies, Hainan Academy of Ocean and Fisheries Sciences, Haikou 571126, China; wangrong1982@gmail.com (R.W.)

* Correspondence: liyuanchaohky@sina.com (Y.L.); hmge@nju.edu.cn (H.G.);guozhikai@itbb.org.cn (Z.G.)

Supporting Information

Figure S1. ^{1}H NMR (500 MHz, acetone-d_6) spectrum of new compound **1**
Figure S2. ^{13}C NMR (125 MHz, acetone-d_6) spectrum of new compound **1**
Figure S3. DEPT135 spectrum of new compound **1**
Figure S4. HSQC spectrum of new compound **1**
Figure S5. HMBC spectrum of new compound **1**
Figure S6. ^{1}H-^{1}H COSY spectrum of new compound **1**
Figure S7. ROESY spectrum of new compound **1**
Figure S8. ^{1}H NMR (600MHz, pyridine-d_5) spectrum of (*R*)-MTPA esters **1**
Figure S9. ^{1}H-^{1}H COSY spectrum of (*R*)-MTPA esters **1**
Figure S10. ^{1}H NMR (600MHz, pyridine-d_5) spectrum of (*S*)-MTPA esters **1**
Figure S11. ^{1}H-^{1}H COSY spectrum of (*S*)-MTPA esters **1**
Figure S12. The HRESIMS spectrum of the new compound **1**
Figure S13. UV spectrum of compound **1**
Figure S14. IR spectrum of compound **1**
Figure S15. ^{1}H NMR (500 MHz, acetone-d_6) spectrum of new compound **2**
Figure S16. ^{13}C NMR (125 MHz, acetone-d_6) spectrum of new compound **2**
Figure S17. DEPT135 spectrum of new compound **2**
Figure S18. HSQC spectrum of new compound **2**
Figure S19. HMBC spectrum of new compound **2**
Figure S20. ^{1}H-^{1}H COSY spectrum of new compound **2**
Figure S21. ROESY spectrum of new compound **2**
Figure S22. ^{1}H NMR (600MHz, pyridine-d_5) spectrum of (*R*)-MTPA esters **2**
Figure S23. ^{1}H-^{1}H COSY spectrum of (*R*)-MTPA esters **2**
Figure S24. ^{1}H NMR (600MHz, pyridine-d_5) spectrum of (*S*)-MTPA esters **2**
Figure S25. ^{1}H-^{1}H COSY spectrum of (*S*)-MTPA esters **2**
Figure S26. The HRESIMS spectrum of the new compound **2**
Figure S27. UV spectrum of compound **2**
Figure S28. IR spectrum of compound **2**
Figure S29. ^{1}H NMR (500 MHz, acetone-d_6) spectrum of new compound **3**
Figure S30. ^{13}C NMR (125 MHz, acetone-d_6) spectrum of new compound **3**
Figure S31. DEPT135 spectrum of new compound **3**
Figure S32. HSQC spectrum of new compound **3**
Figure S33. HMBC spectrum of new compound **3**
Figure S34. ^{1}H-^{1}H COSY spectrum of new compound **3**
Figure S35. ROESY spectrum of new compound **3**
Figure S36. The HRESIMS spectrum of the new compound **3**
Figure S37. UV spectrum of compound **3**
Figure S38. IR spectrum of compound **3**
Figure S39. ^{1}H NMR (500 MHz, acetone-d_6) spectrum of new compound **4**
Figure S40. ^{13}C NMR (125 MHz, acetone-d_6) spectrum of new compound **4**
Figure S41. DEPT135 spectrum of new compound **4**
Figure S42. HSQC spectrum of new compound **4**

Figure S43. HMBC spectrum of new compound **4**
Figure S44. ^{1}H-^{1}H COSY spectrum of new compound **4**
Figure S45. ROESY spectrum of new compound **4**
Figure S46. The HRESIMS spectrum of new compound **4**
Figure S47. UV spectrum of compound **4**
Figure S48. IR spectrum of compound **4**
Figure S49. The ECD spectra of compounds **1-3**

Figure S1. ^{1}H NMR (500 MHz, acetone-d_6) spectrum of new compound **1**

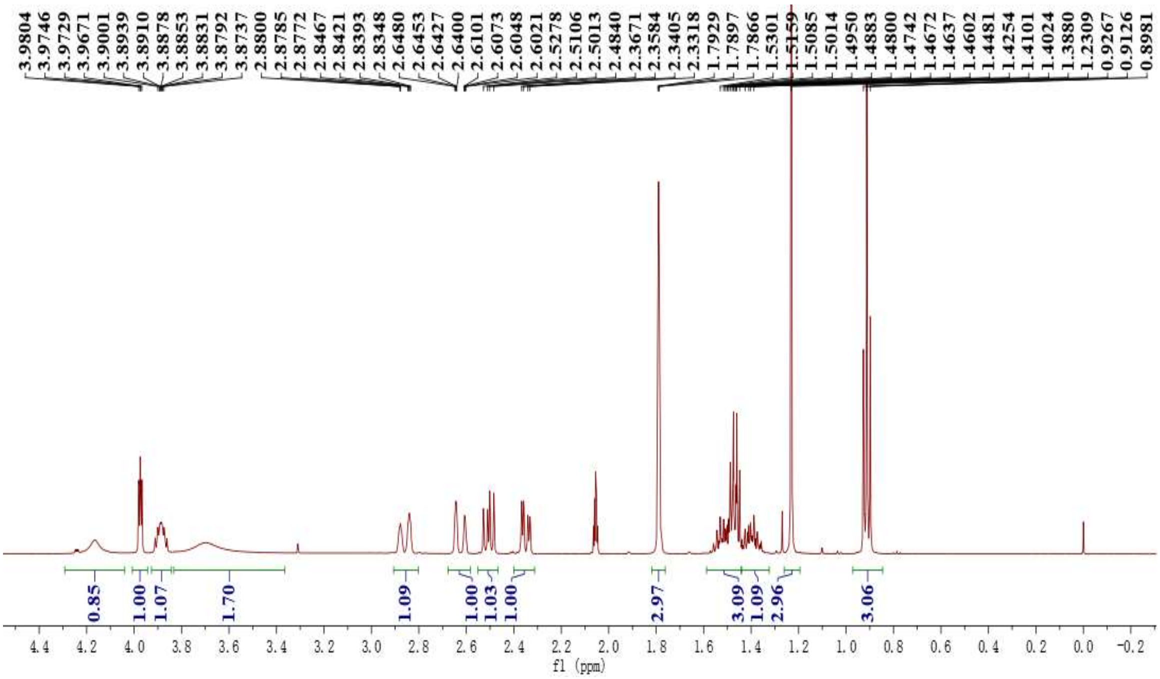

Figure S2. ^{13}C NMR (125 MHz, acetone-d_6) spectrum of new compound **1**

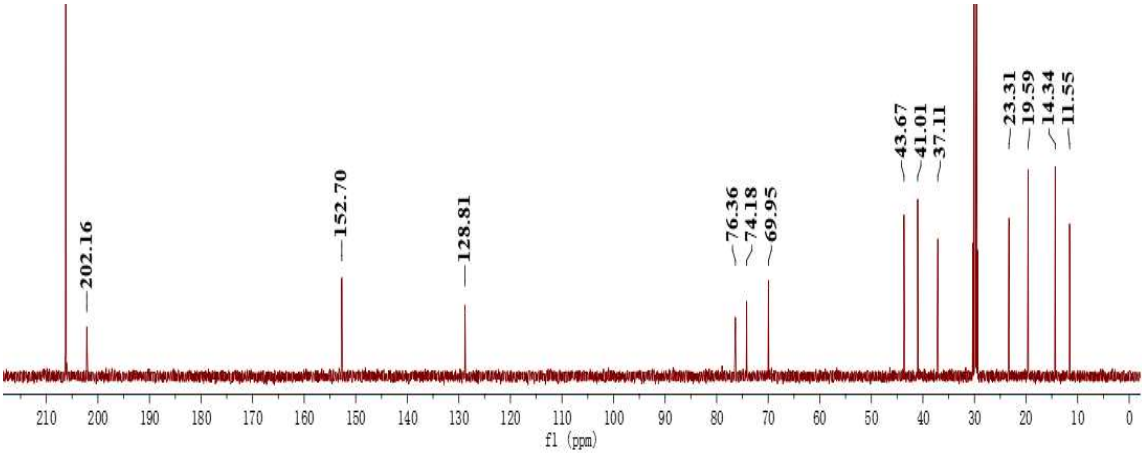

Figure S3. DEPT135 spectrum of new compound **1**

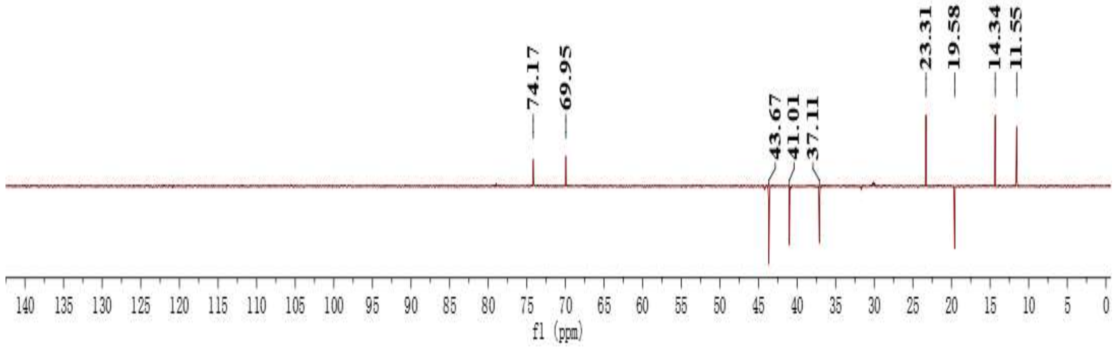

Figure S4. HSQC spectrum of new compound **1**

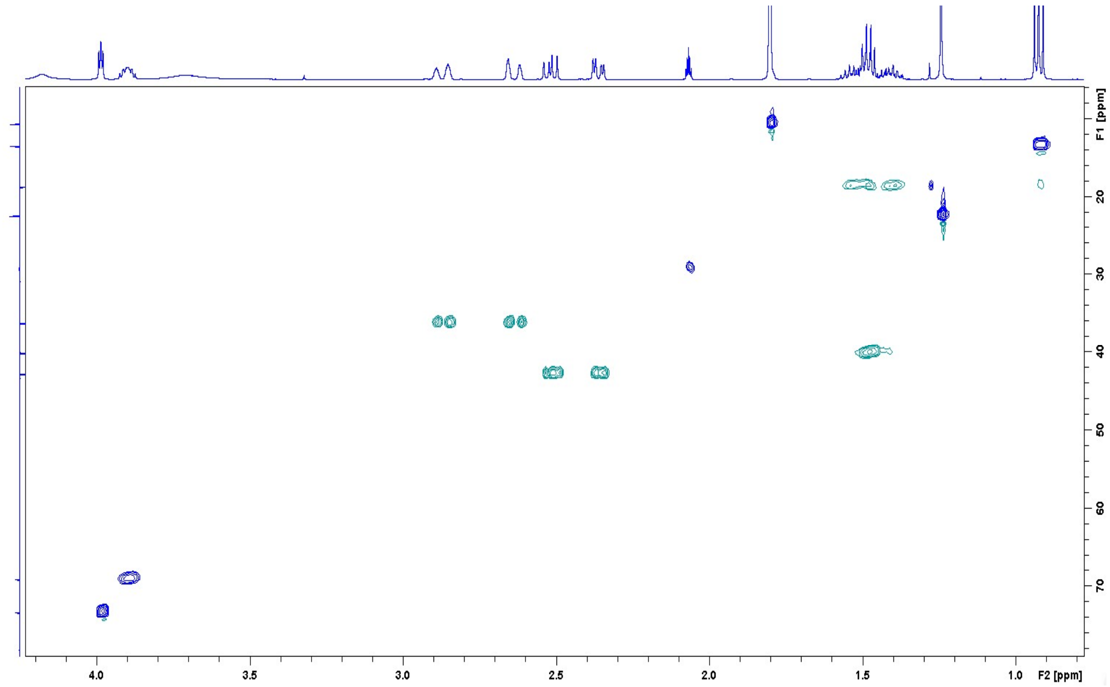

Figure S5. HMBC spectrum of new compound **1**

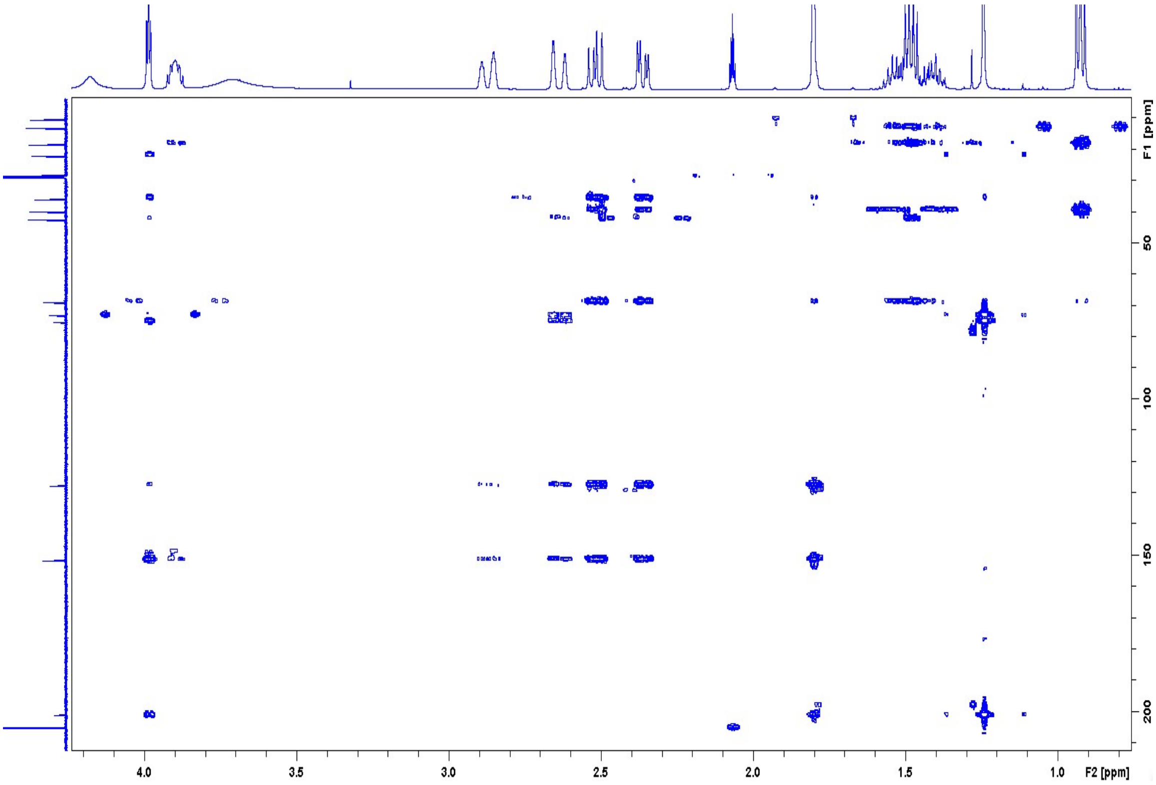

Figure S6. ^{1}H-^{1}H COSY spectrum of new compound **1**

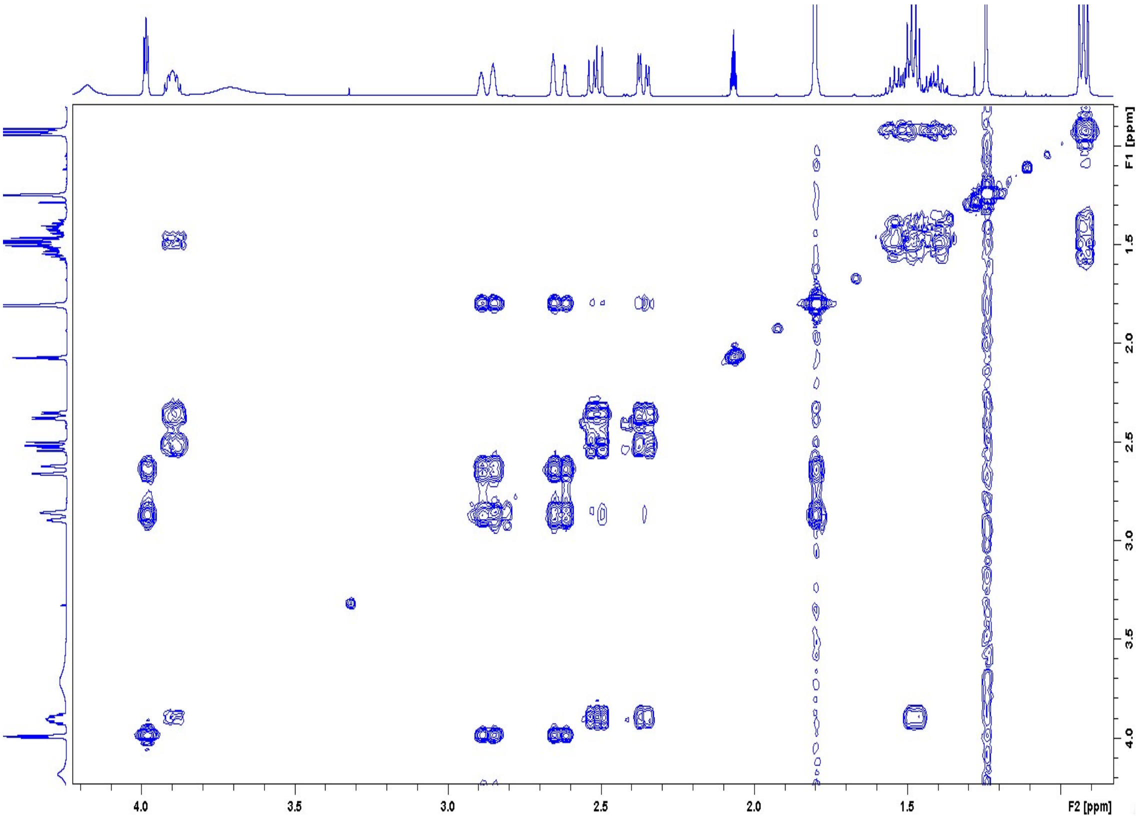

Figure S7. ROESY spectrum of new compound **1**

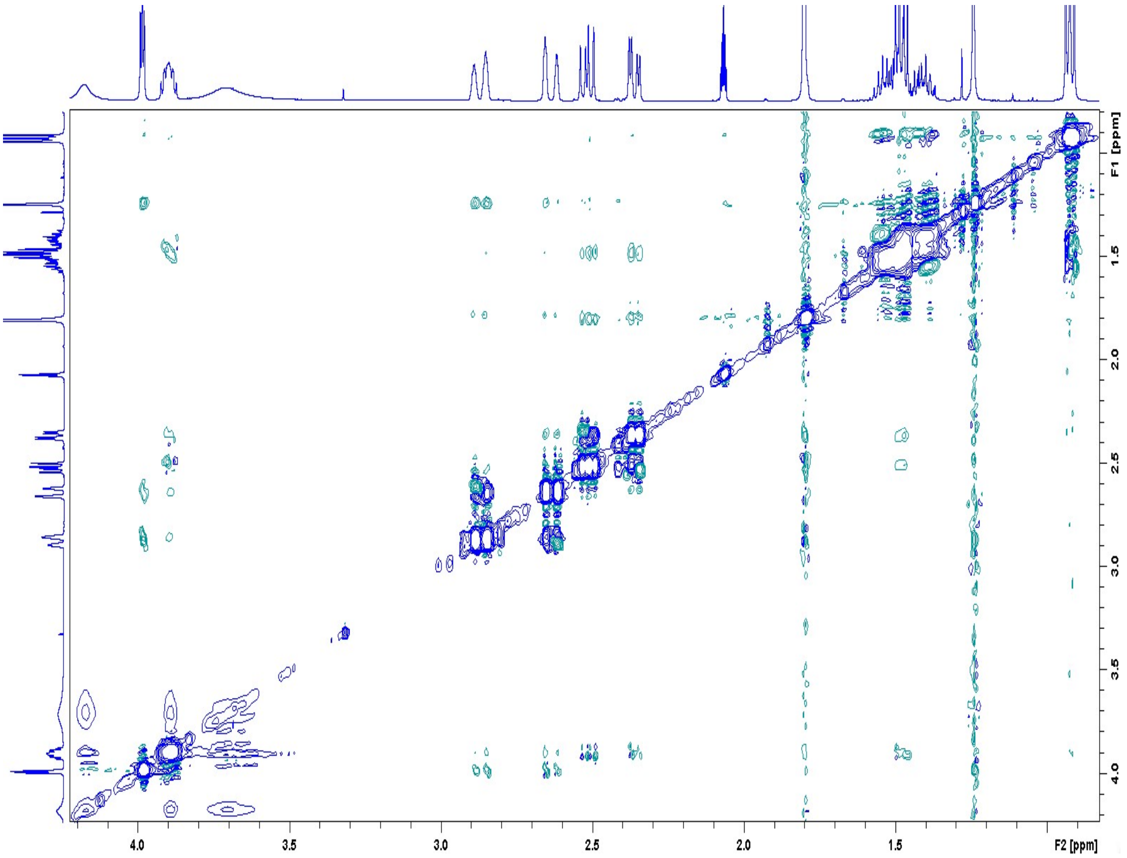

Figure S8. ^{1}H NMR (600MHz, pyridine-d_5) spectrum of (*R*)-MTPA esters **1**

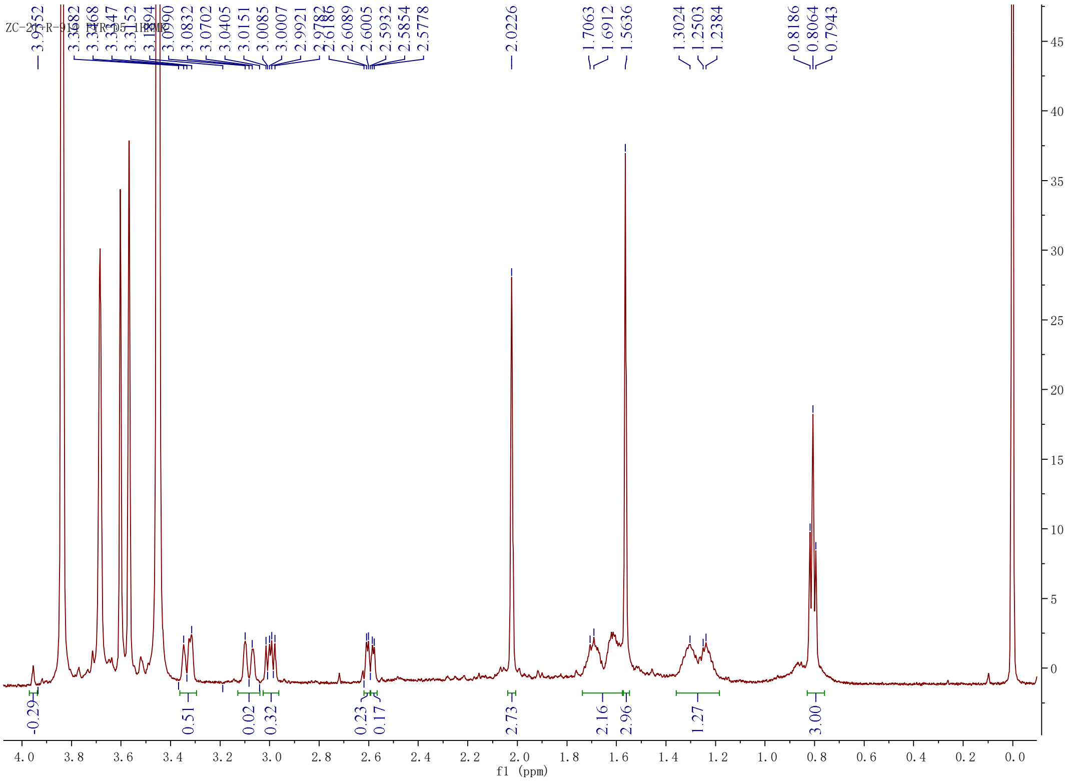

Figure S9. ^{1}H-^{1}H COSY spectrum of (*R*)-MTPA esters **1**

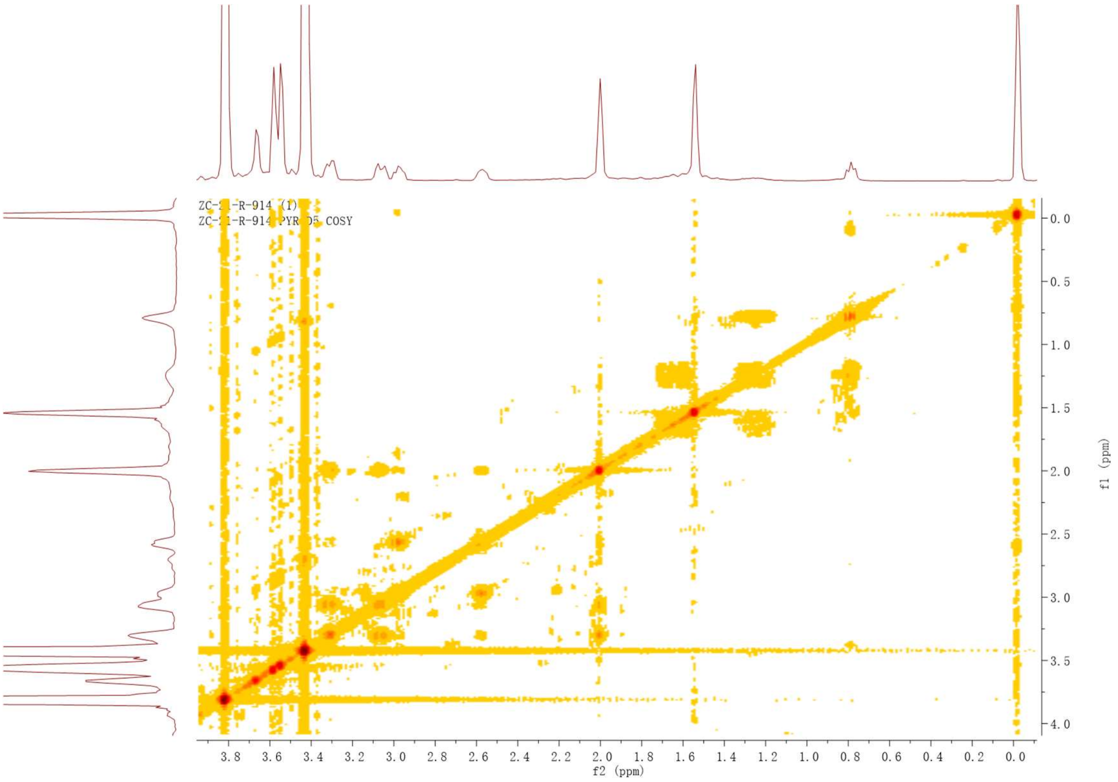

Figure S10. ^{1}H NMR (600MHz, pyridine-d_5) spectrum of (*S*)-MTPA esters **1**

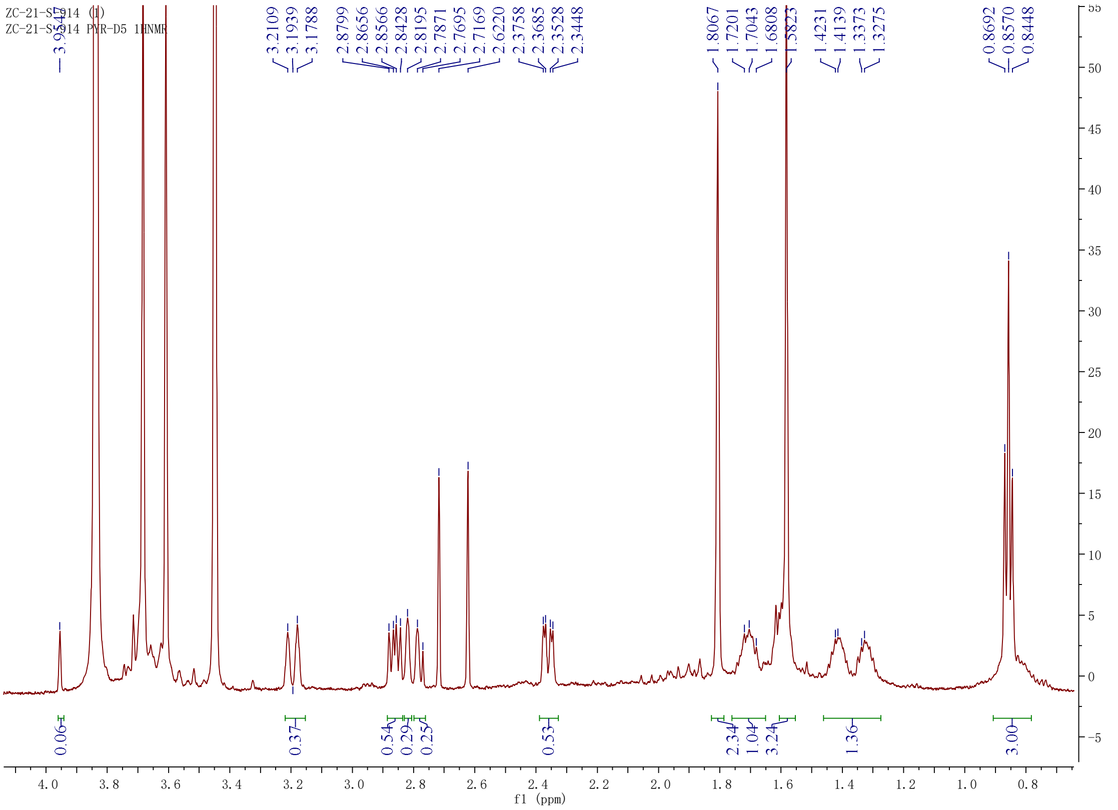

Figure S11. ^{1}H-^{1}H COSY spectrum of (*S*)-MTPA esters **1**

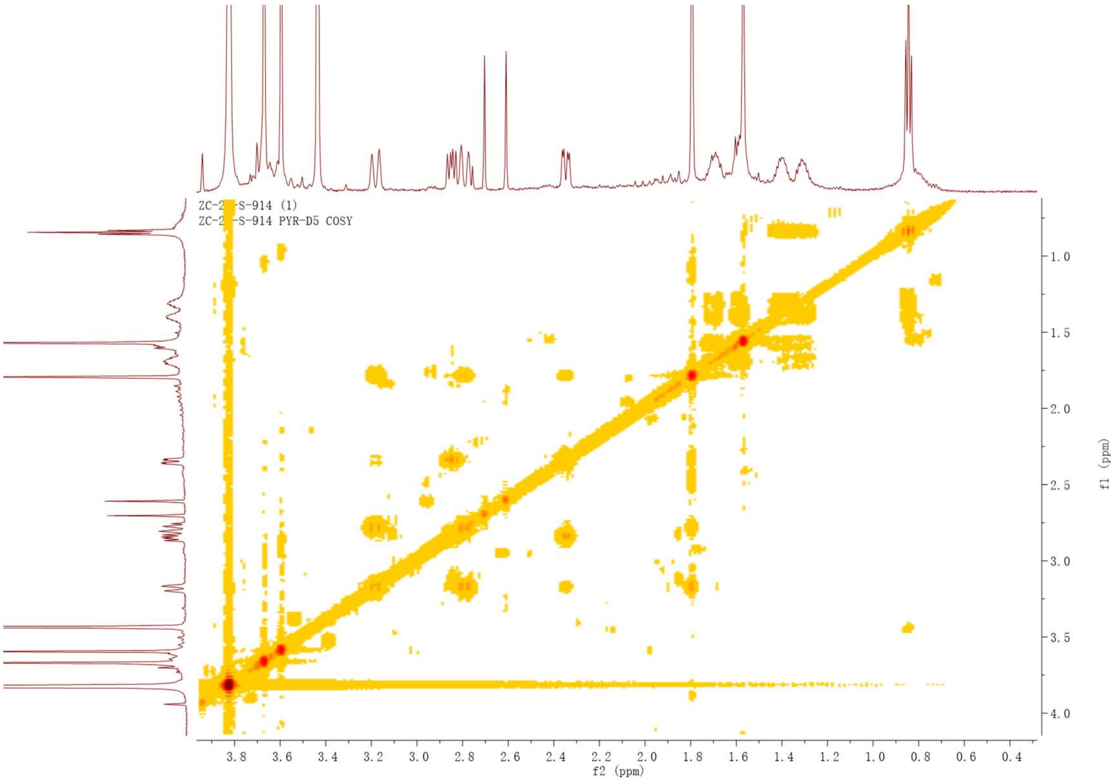

Figure S12. The HRESIMS spectrum of the new compound **1**

m / z 265.1410 $[M+Na]^+$ (calcd for $C_{13}H_{22}NaO_4$, 265.1410)

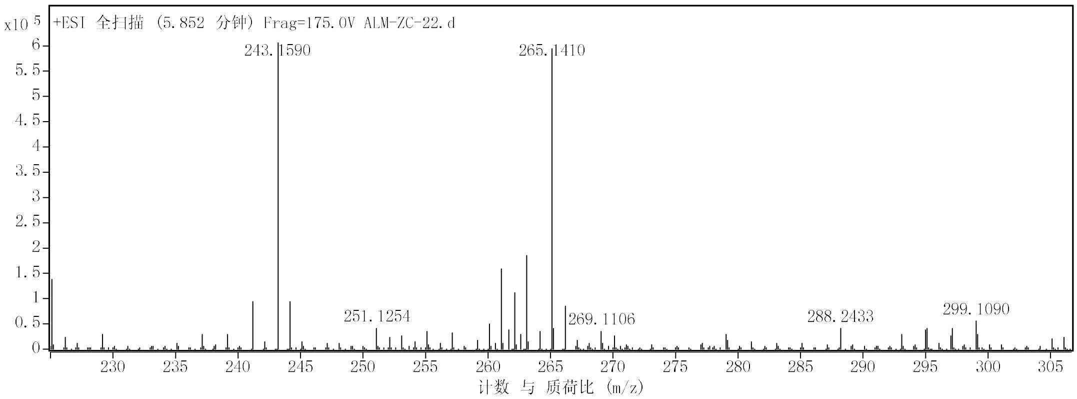

Figure S13. UV spectrum of compound **1**

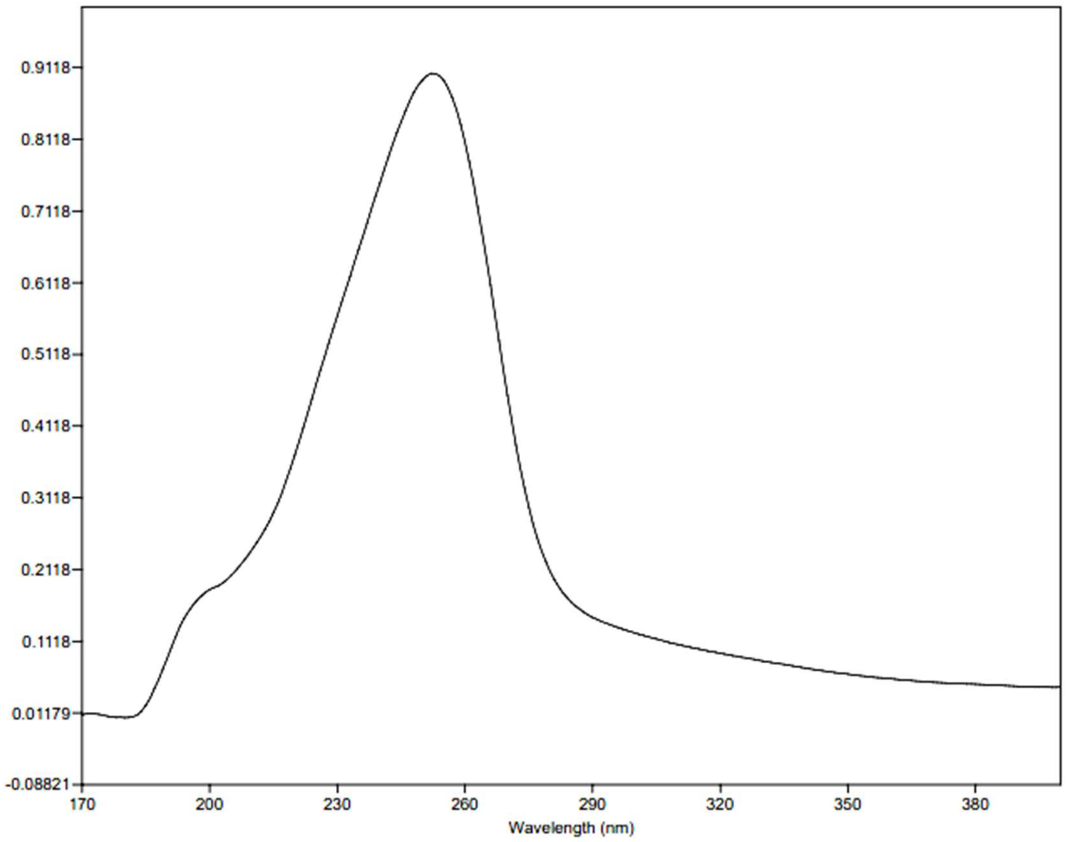

Figure S14. IR spectrum of compound **1**

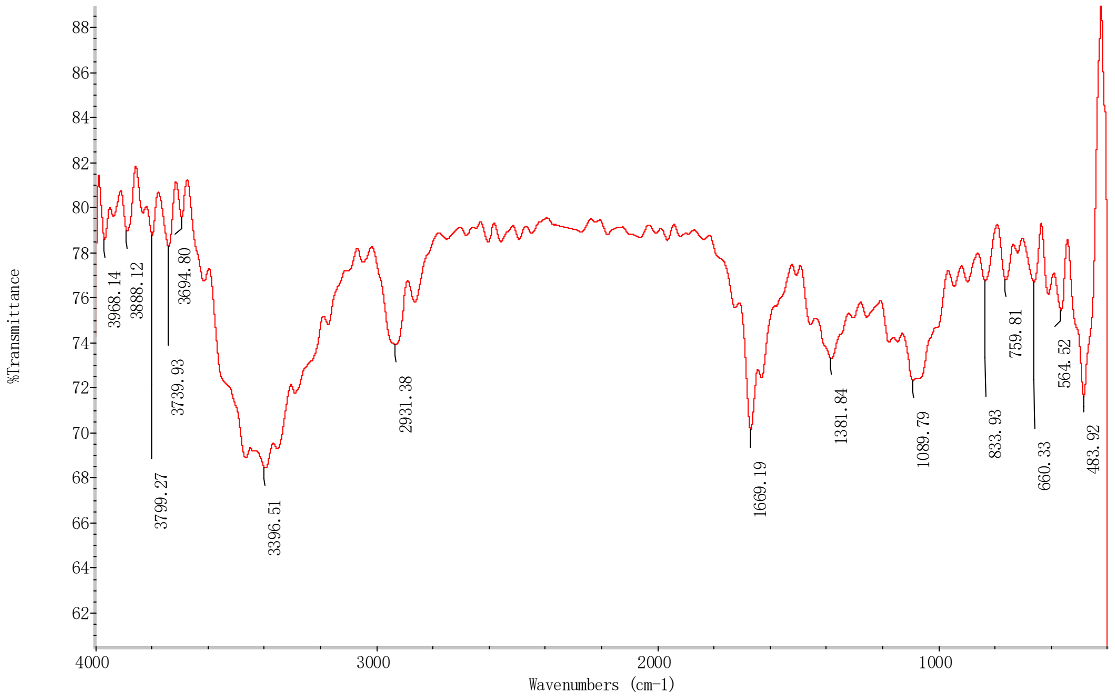

Figure S15. ^{1}H NMR (500 MHz, acetone-d_6) spectrum of new compound **2**

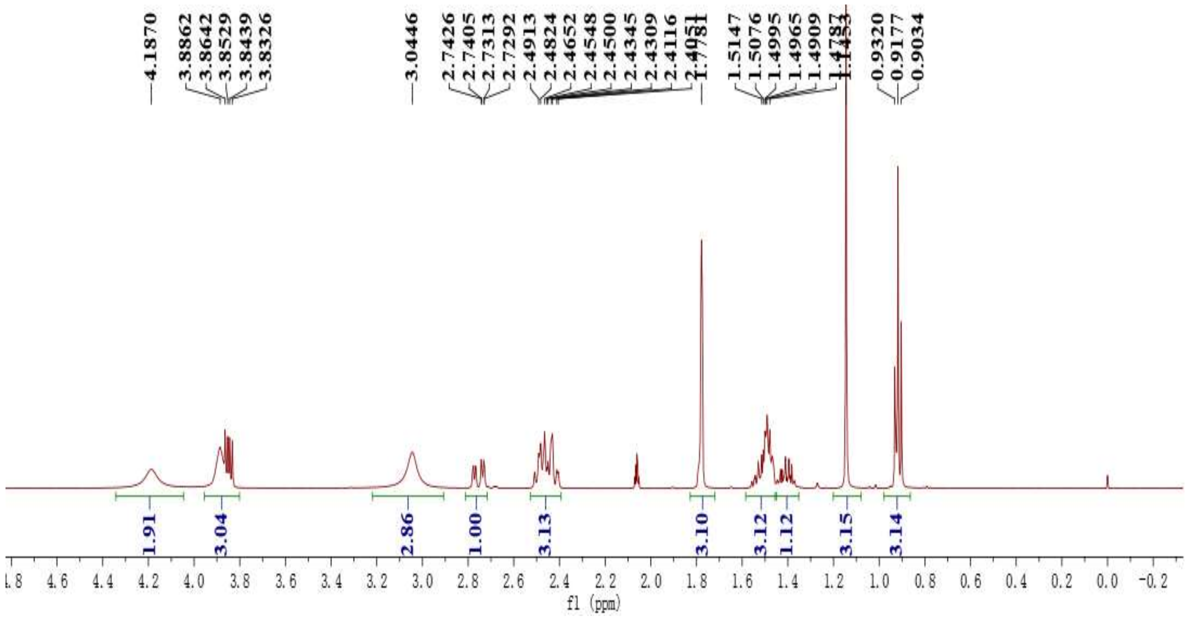

Figure S16. ^{13}C NMR (125 MHz, acetone-d_6) spectrum of new compound **2**

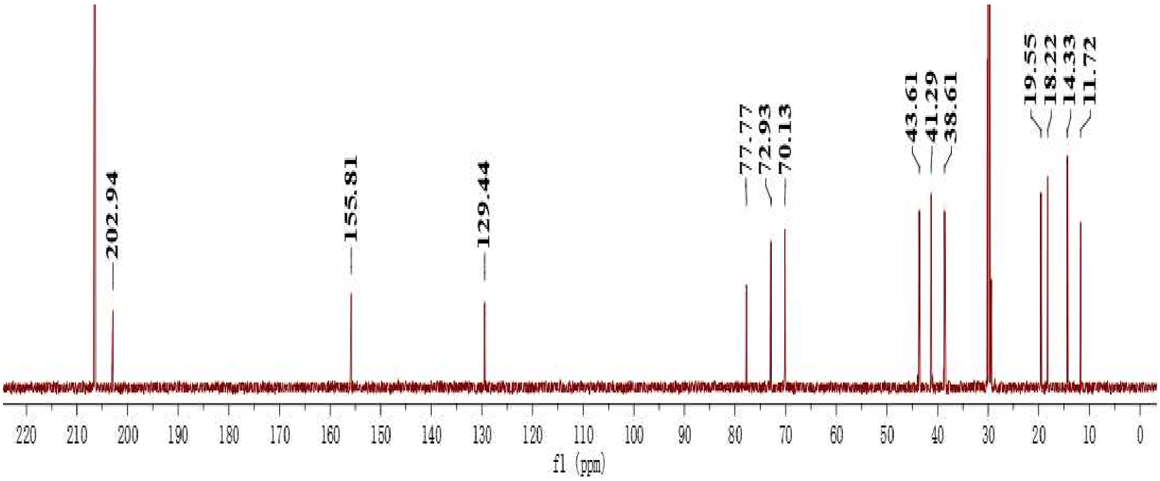

Figure S17. DEPT135 spectrum of new compound **2**

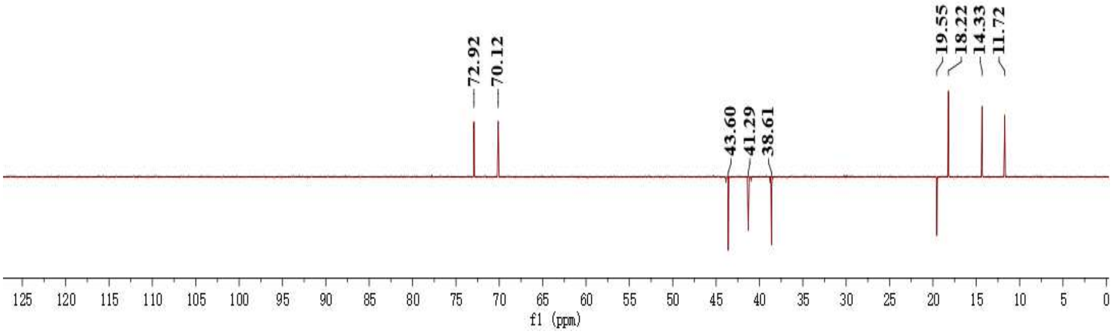

Figure S18. HSQC spectrum of new compound **2**

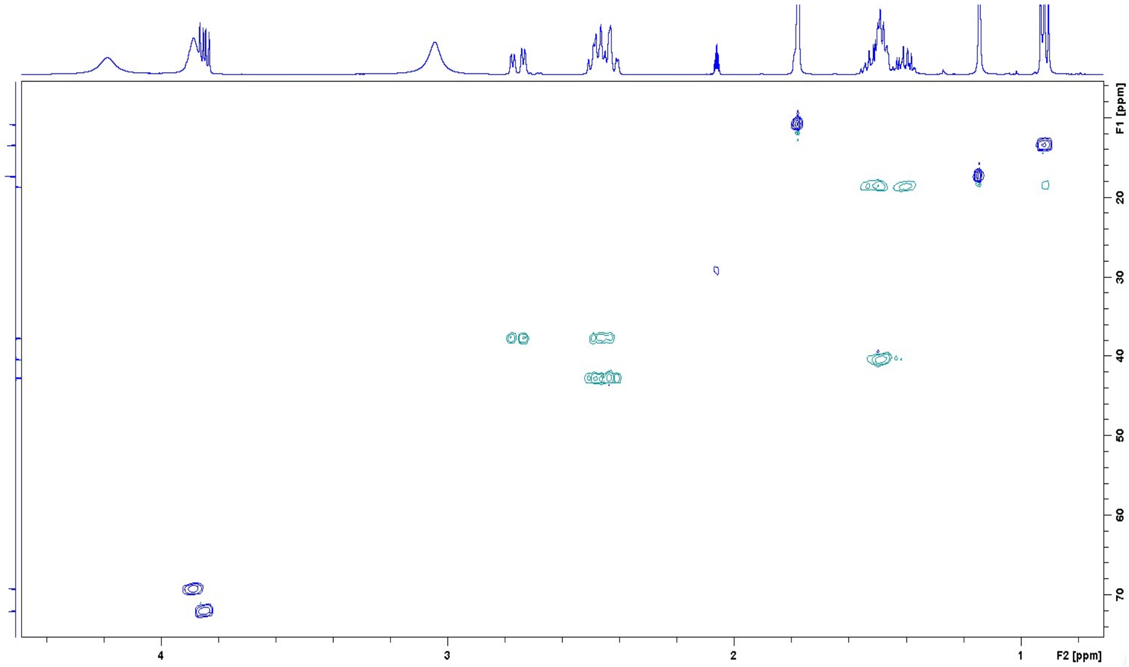

Figure S19. HMBC spectrum of new compound **2**

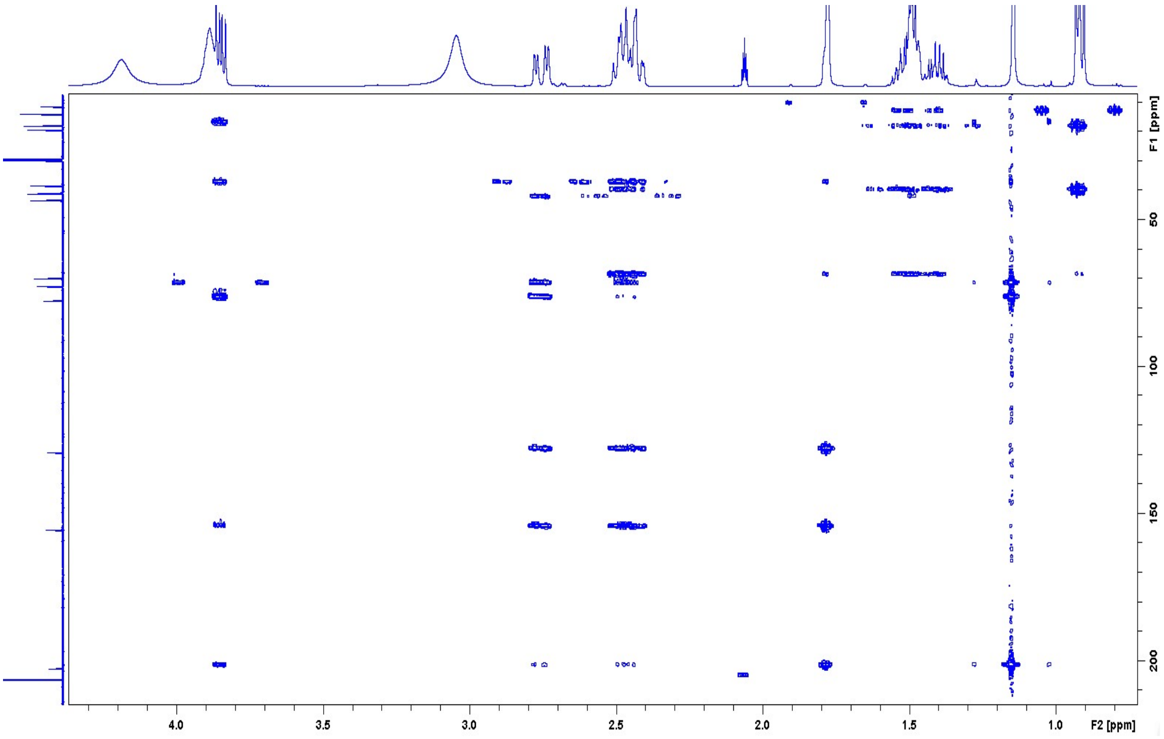

Figure S20. ^{1}H-^{1}H COSY spectrum of new compound **2**

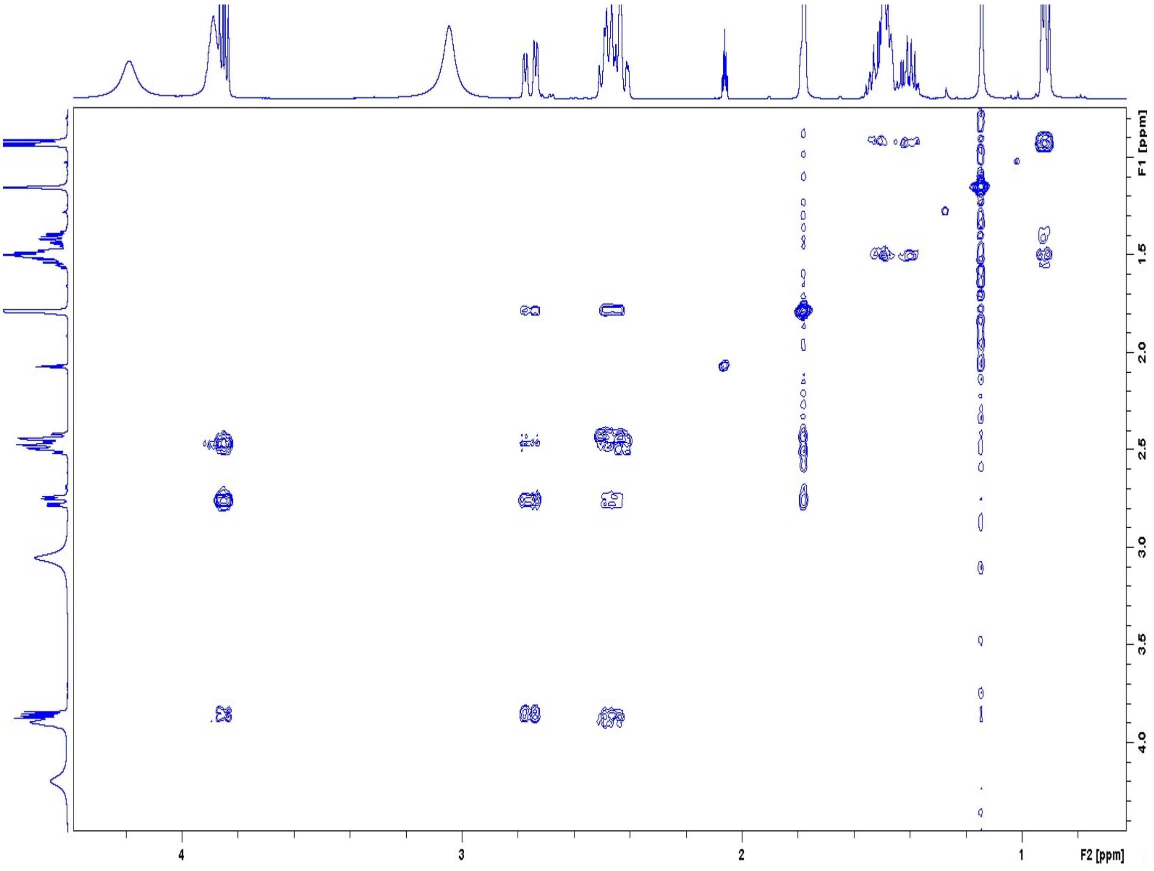

Figure S21. ROESY spectrum of new compound **2**

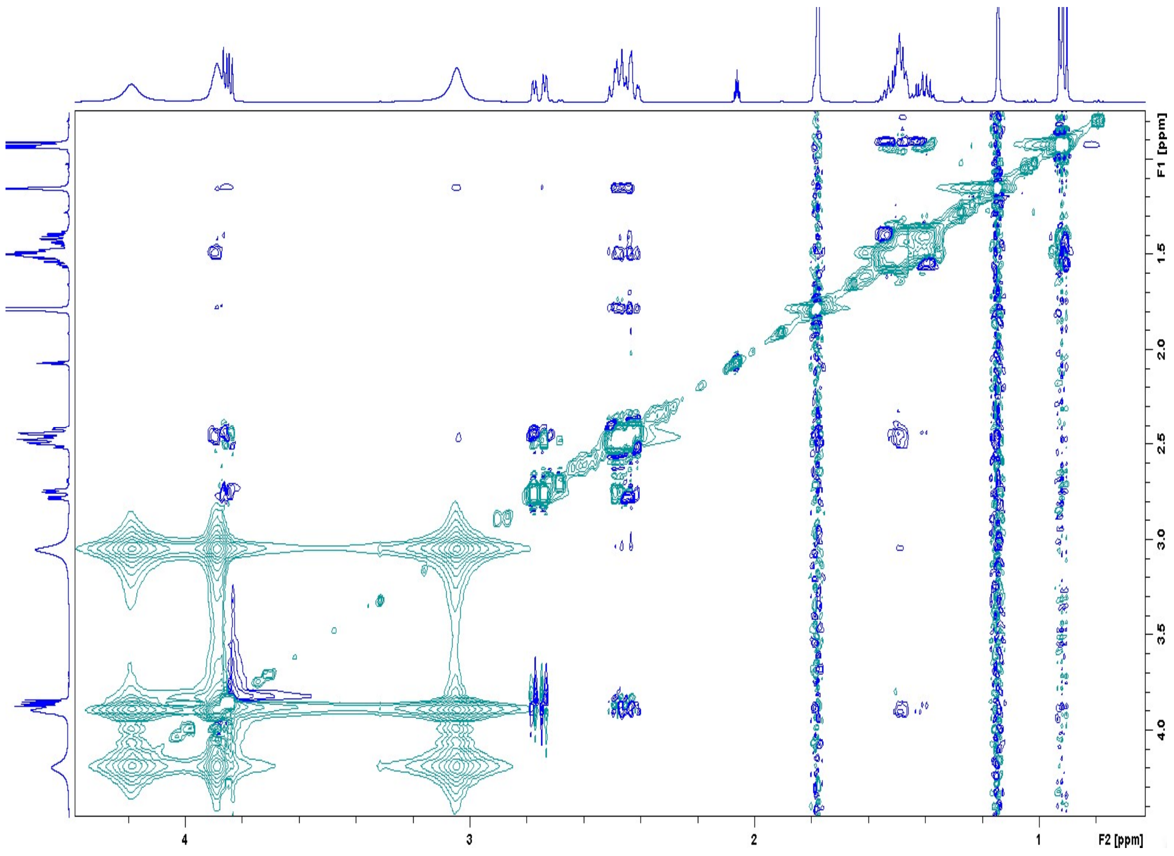

Figure S22. ^{1}H NMR (600MHz, pyridine-d_5) spectrum of (R)-MTPA esters **2**

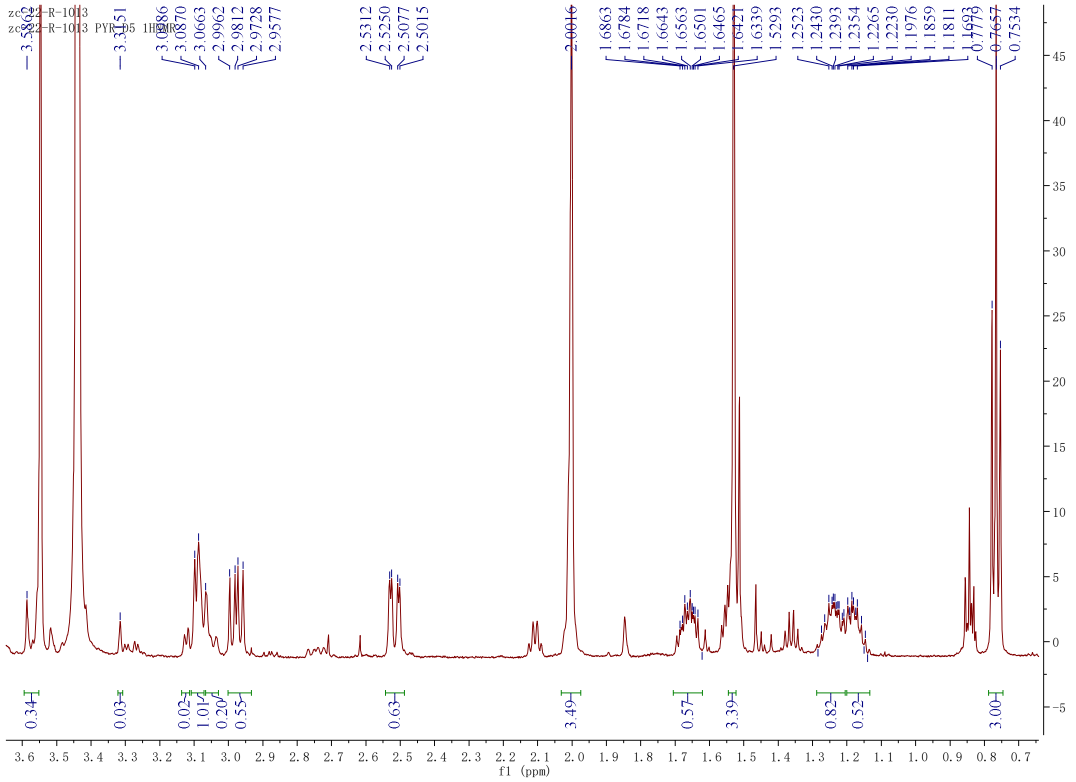

Figure S23. ^{1}H-^{1}H COSY spectrum of (R)-MTPA esters **2**

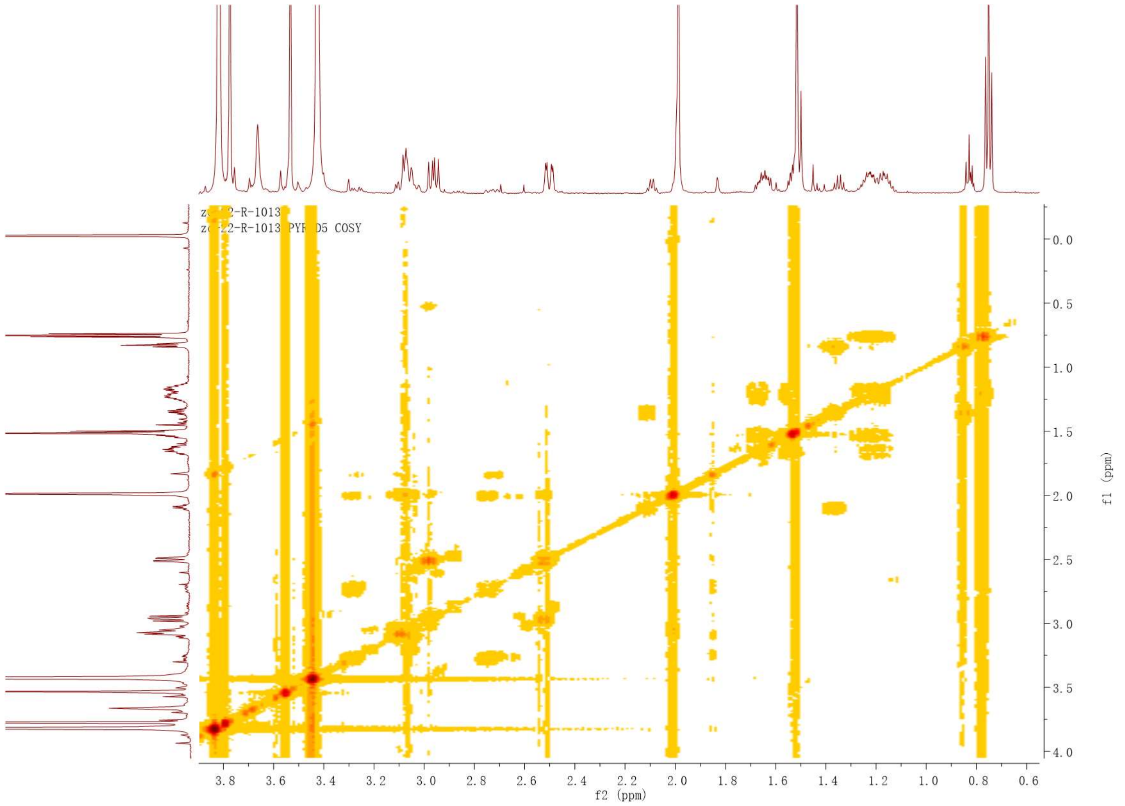

Figure S24. ^{1}H NMR (600MHz, pyridine-d_5) spectrum of (*S*)-MTPA esters **2**

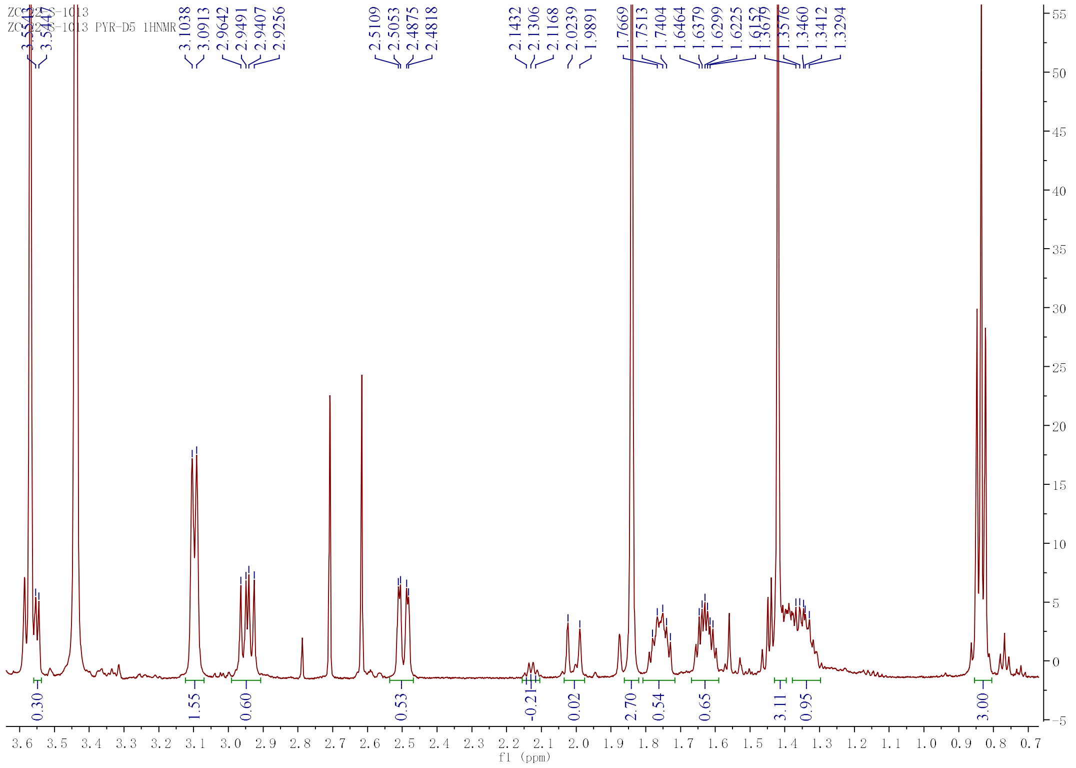

Figure S25. ^{1}H-^{1}H COSY spectrum of (*S*)-MTPA esters **2**

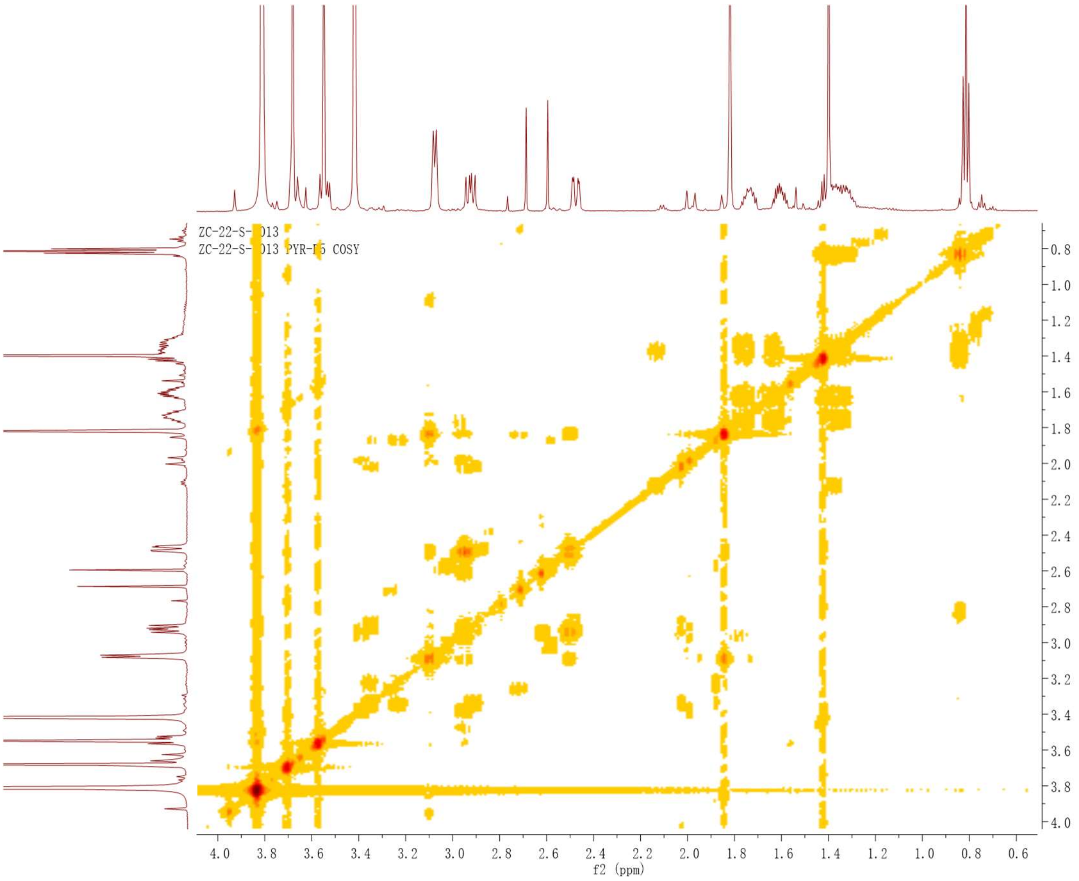

Figure S26. The HRESIMS spectrum of the new compound **2**

m / z 265.1447 $[M+Na]^+$ (calcd for $C_{13}H_{22}NaO_4$, 265.1410)

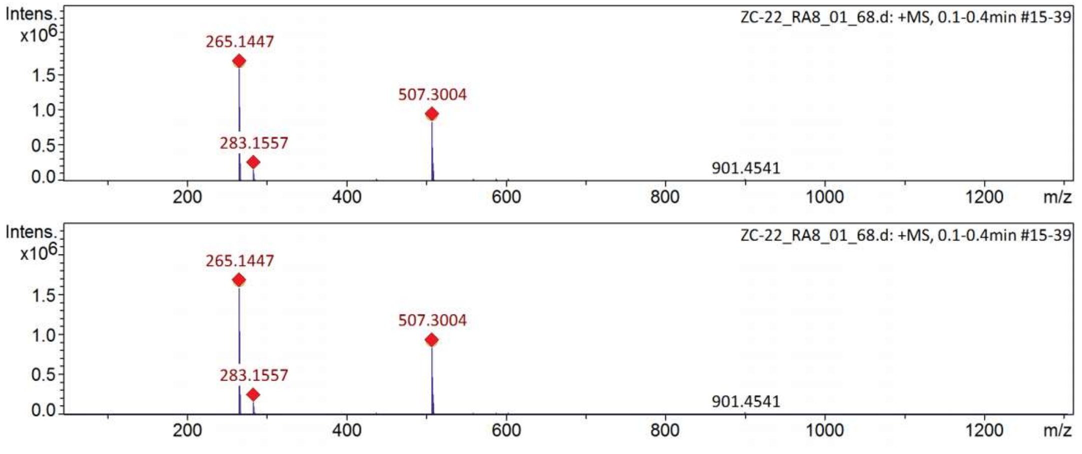

Meas. m/z	#	Ion Formula	m/z	err [ppm]	mSigma	# mSigma	Score	rdb	e⁻ Conf	N-Rule	Adduct
265.1447	1	C13H22NaO4	265.1410	-14.0	1.6	1	100.00	3.0	even	ok	M+Na

Figure S27. UV spectrum of compound **2**

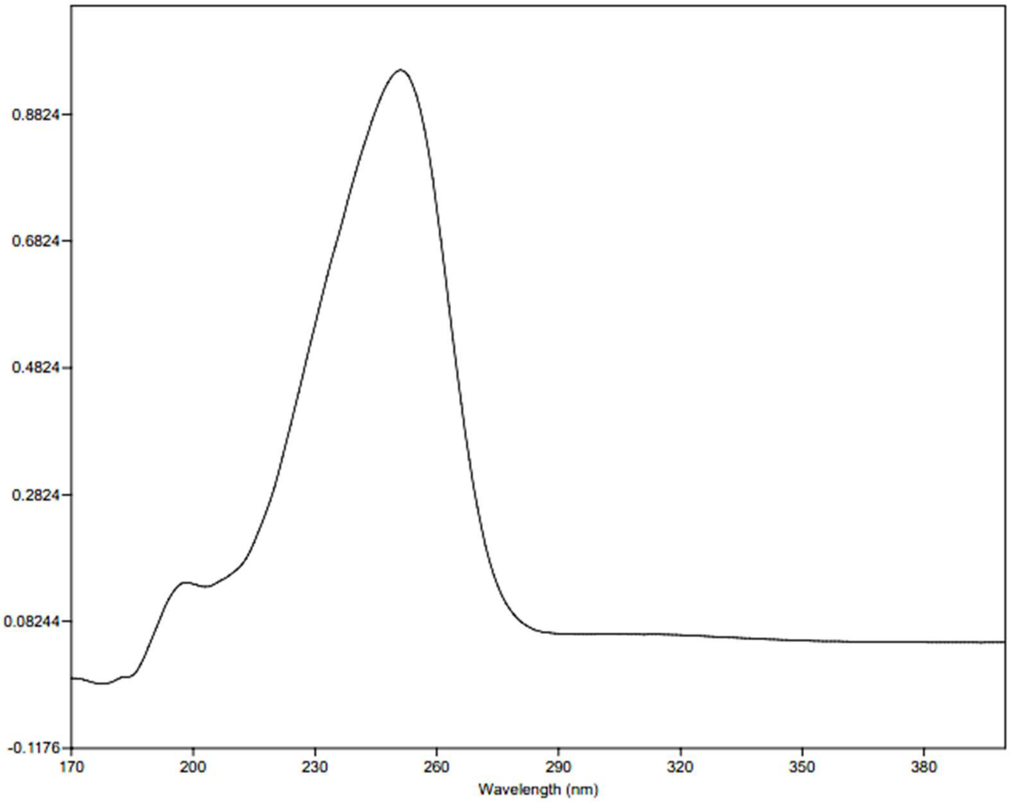

Figure S28. IR spectrum of compound **2**

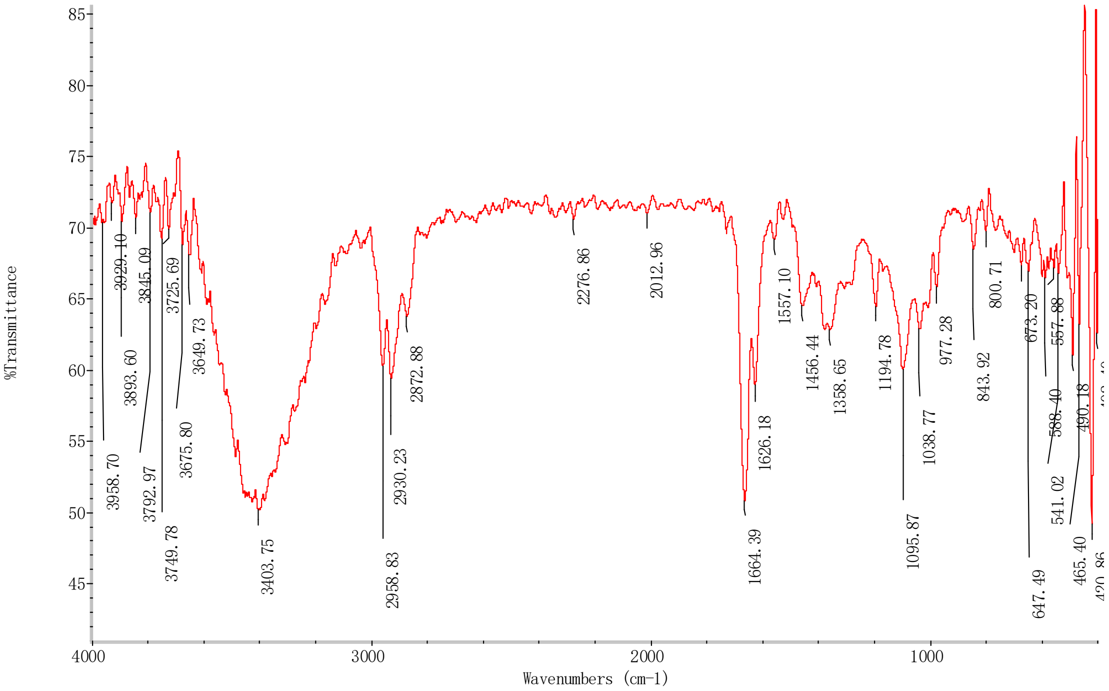

Figure S29. ^{1}H NMR (500 MHz, acetone-d_6) spectrum of new compound **3**

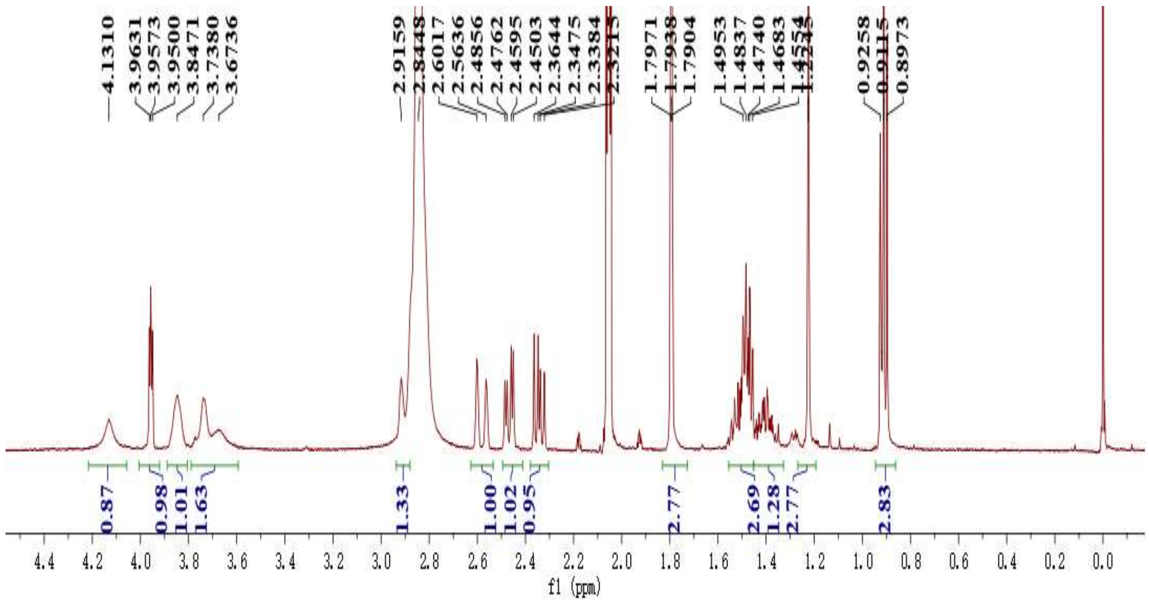

Figure S30. ^{13}C NMR (125 MHz, acetone-d_6) spectrum of new compound **3**

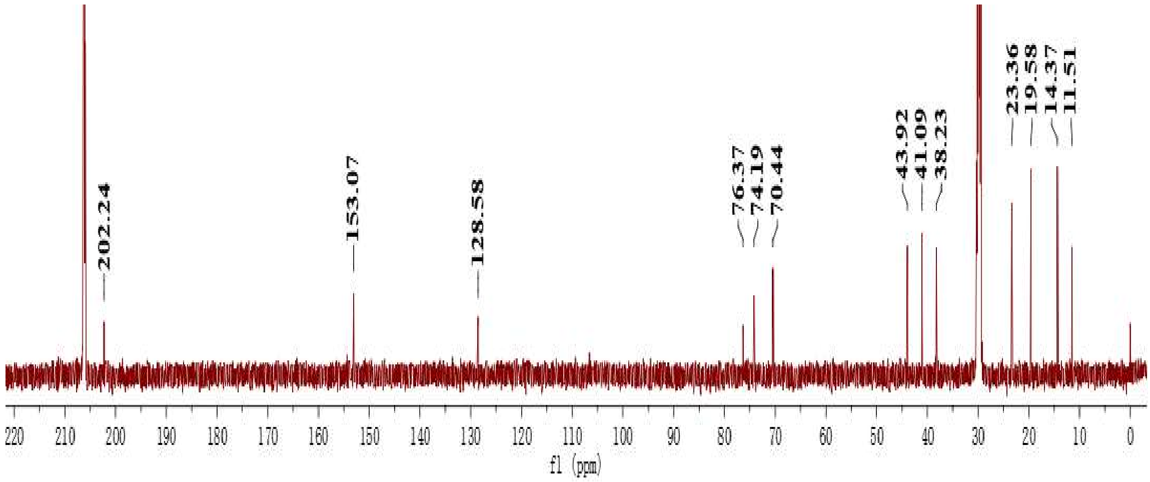

Figure S31. DEPT135 spectrum of new compound **3**

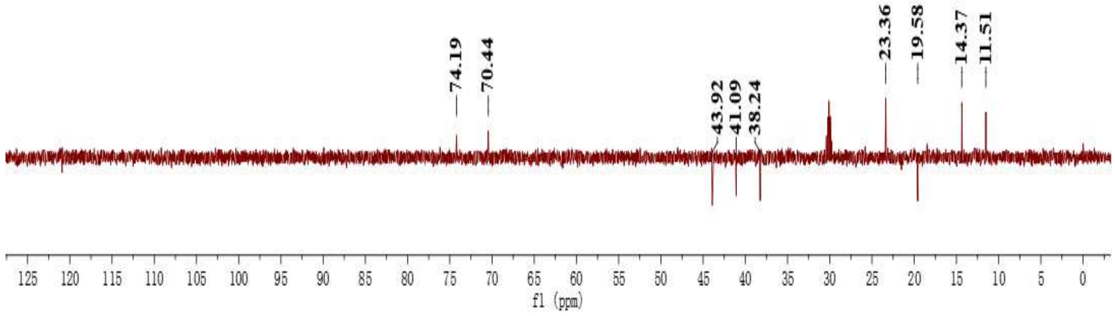

Figure S32. HSQC spectrum of new compound **3**

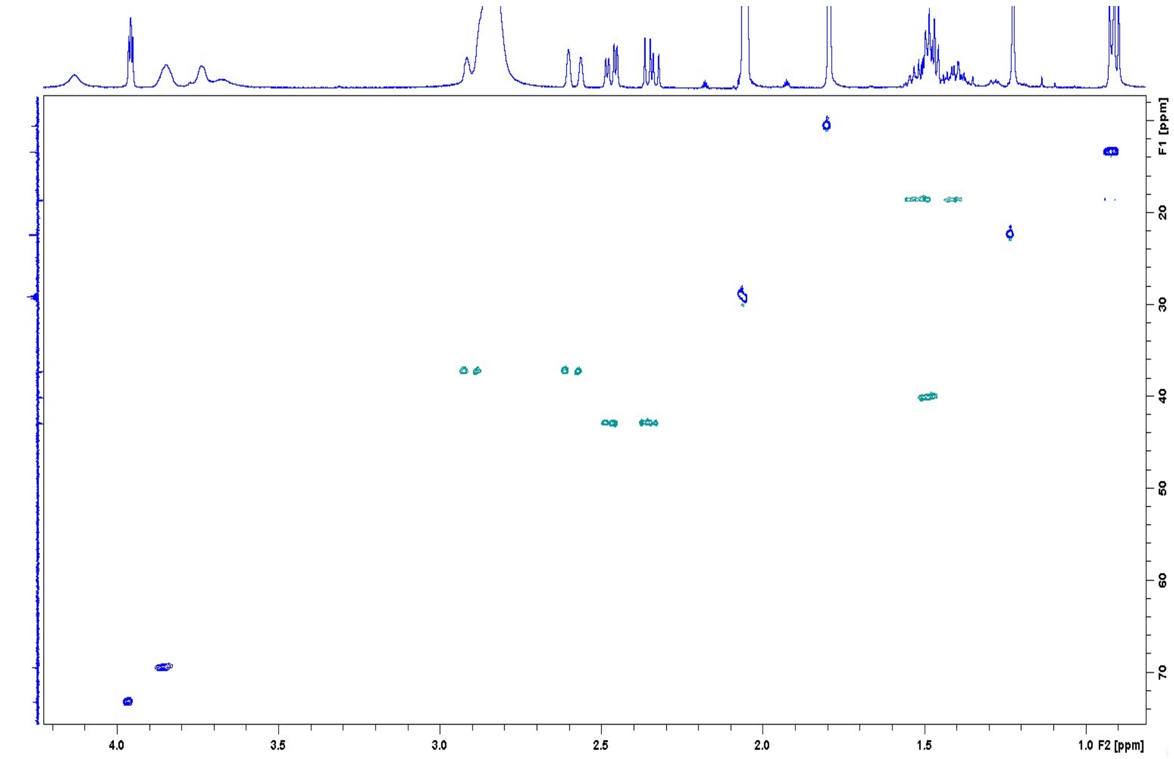

Figure S33. HMBC spectrum of new compound **3**

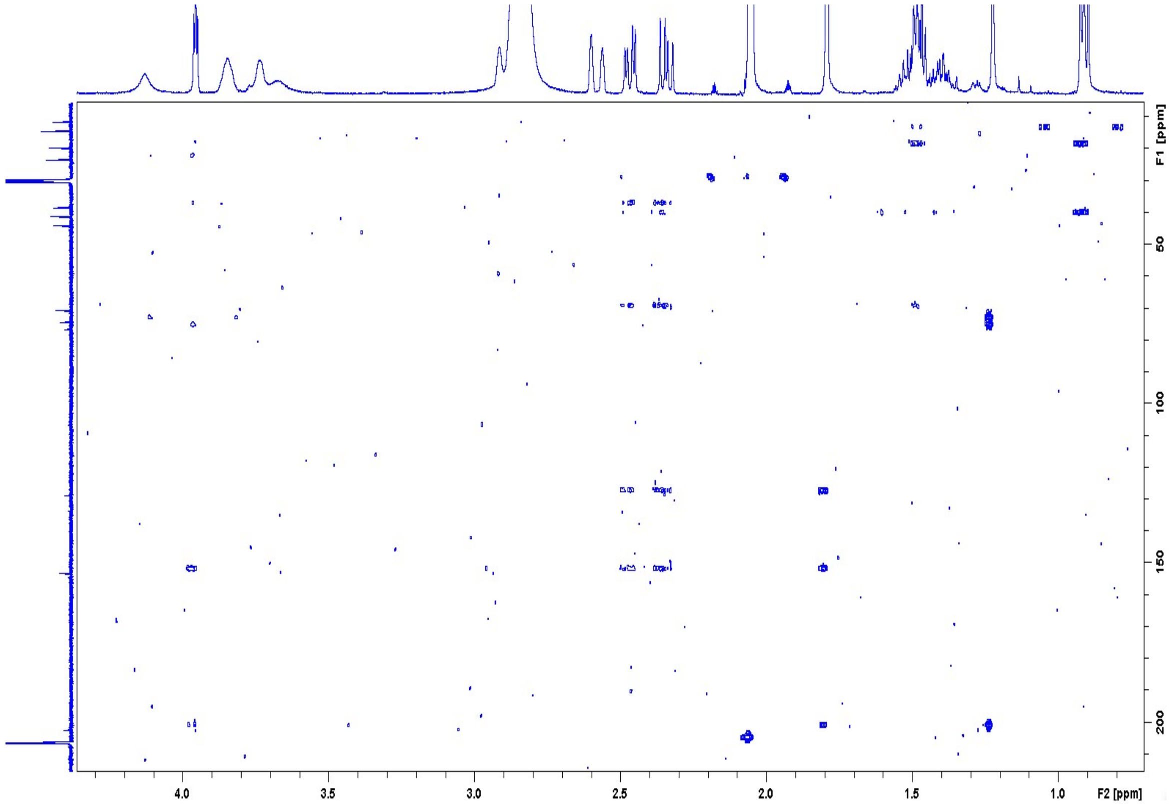

Figure S34. ^{1}H-^{1}H COSY spectrum of new compound **3**

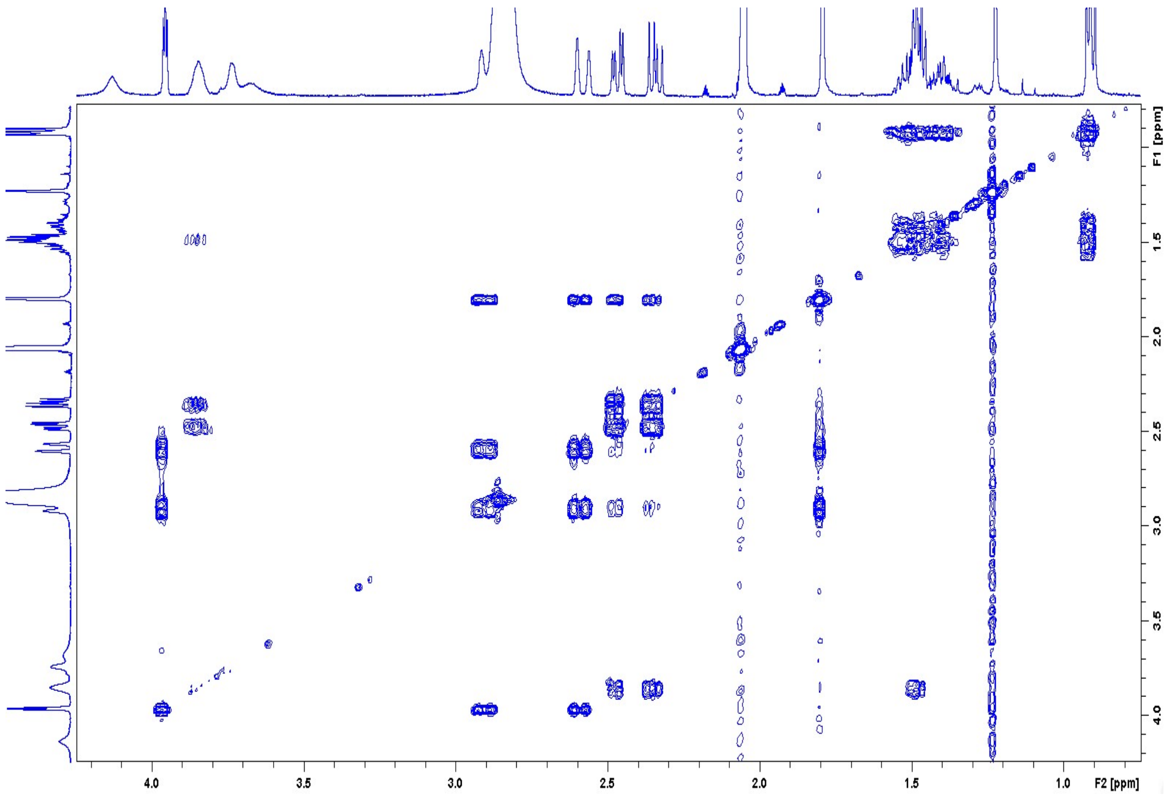

Figure S35. ROESY spectrum of new compound **3**

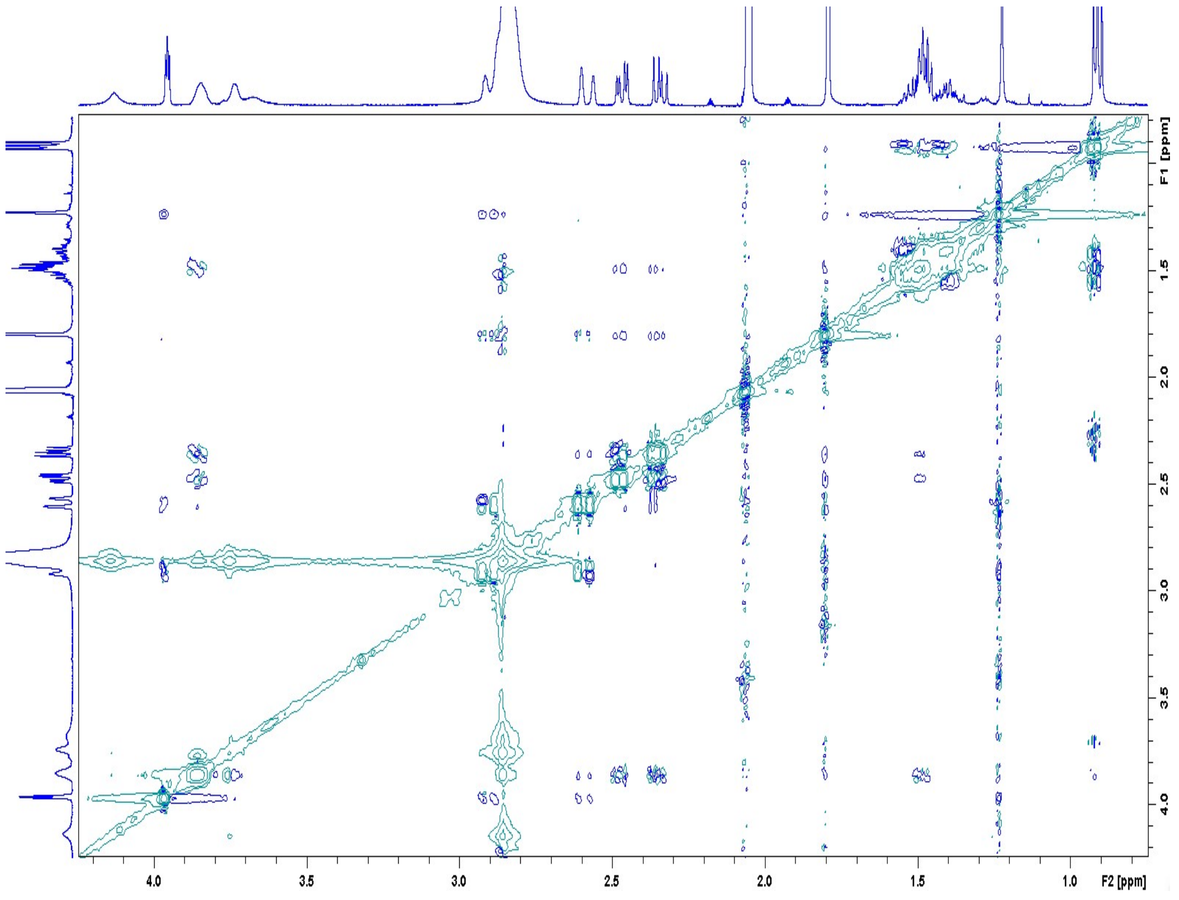

Figure S36. The HRESIMS spectrum of the new compound **3**

m / z 243.1595 $[M+H]^+$ (calcd for $C_{13}H_{23}O_4$, 243.1591)

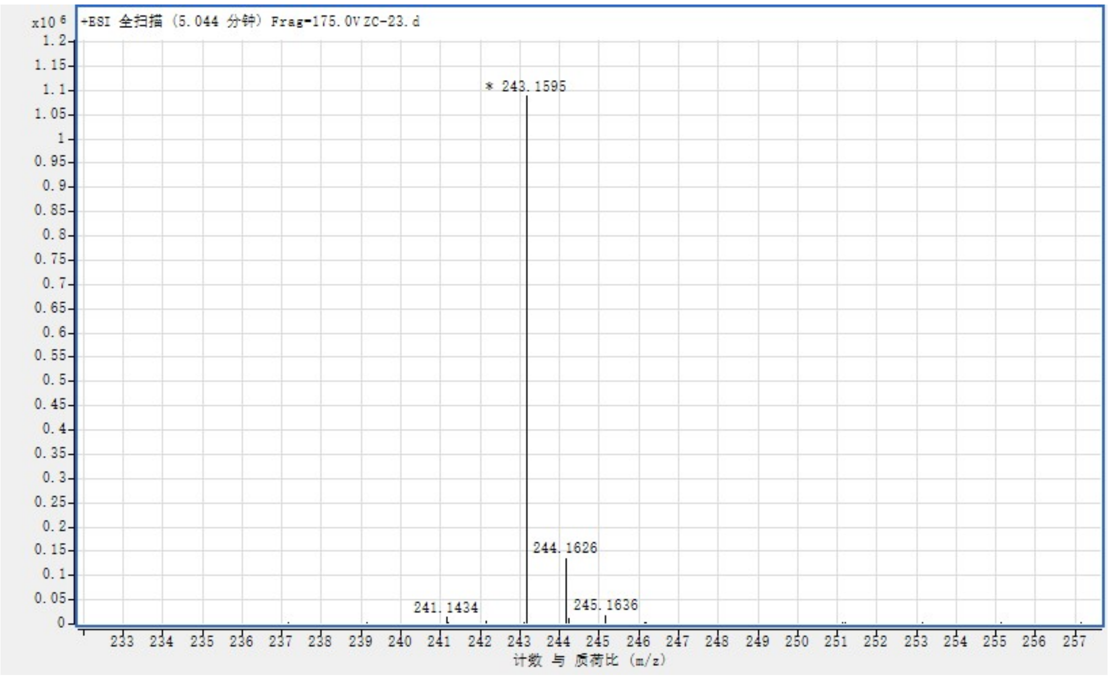

Figure S37. UV spectrum of compound **3**

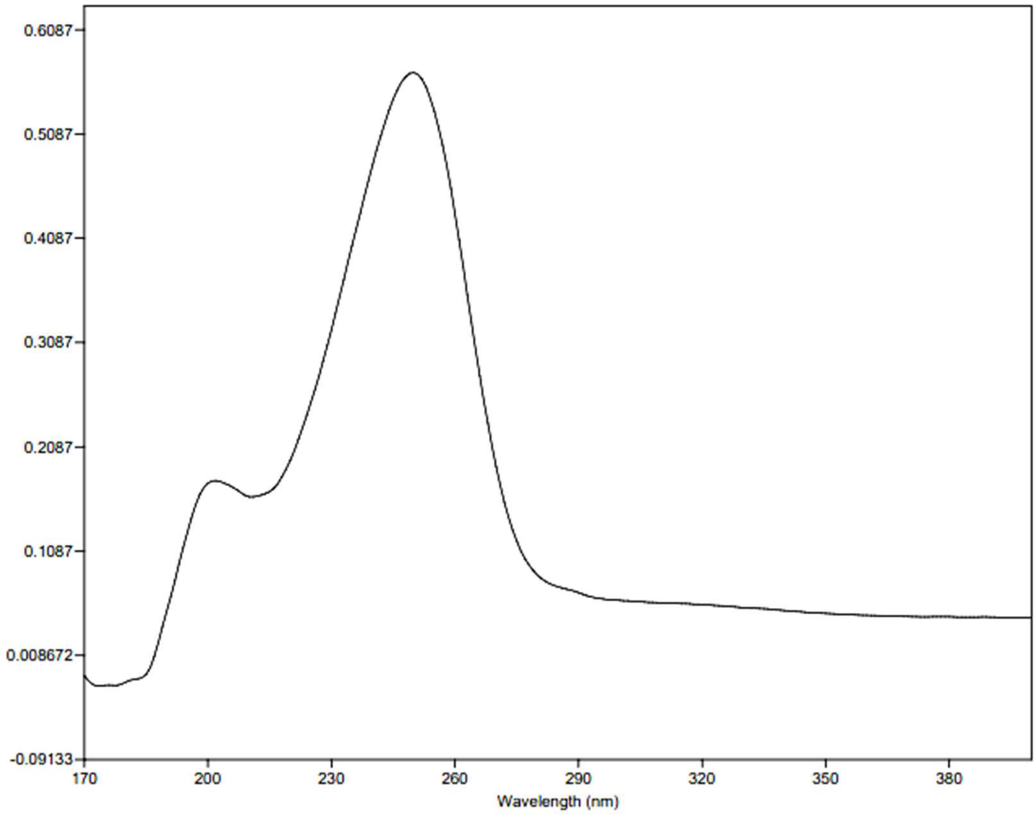

Figure S38. IR spectrum of compound **3**

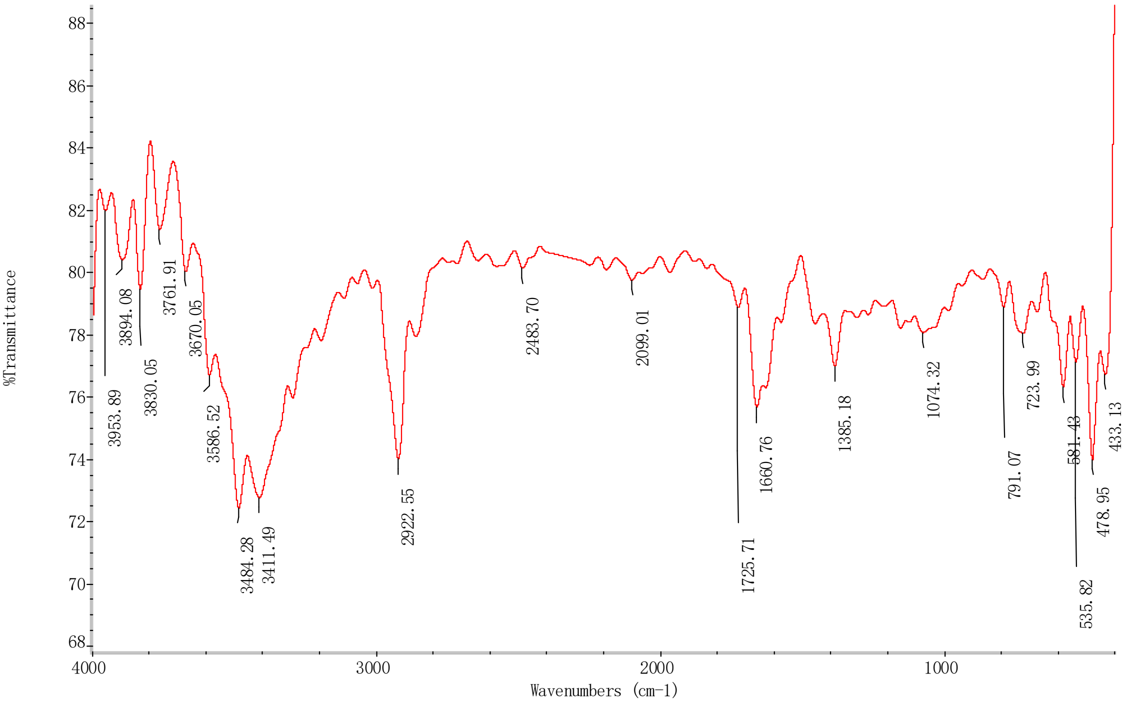

Figure S39. ^{1}H NMR (500 MHz, acetone-d_6) spectrum of new compound **4**

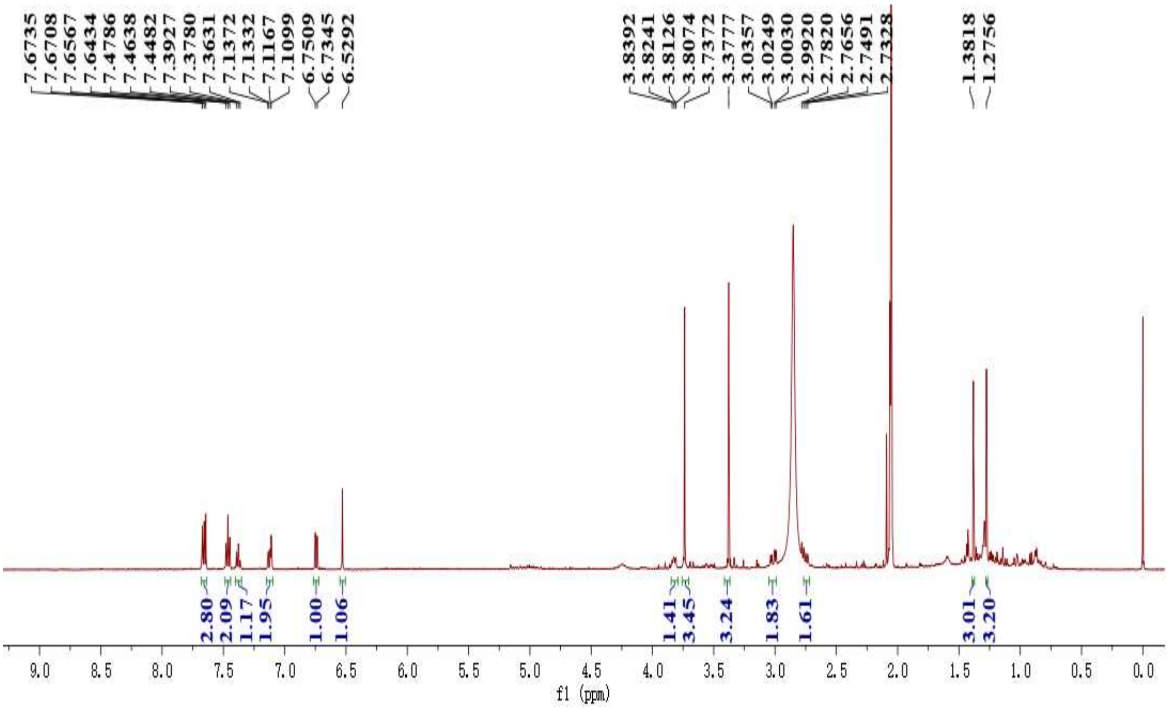

Figure S40. ^{13}C NMR (125 MHz, acetone-d_6) spectrum of new compound **4**

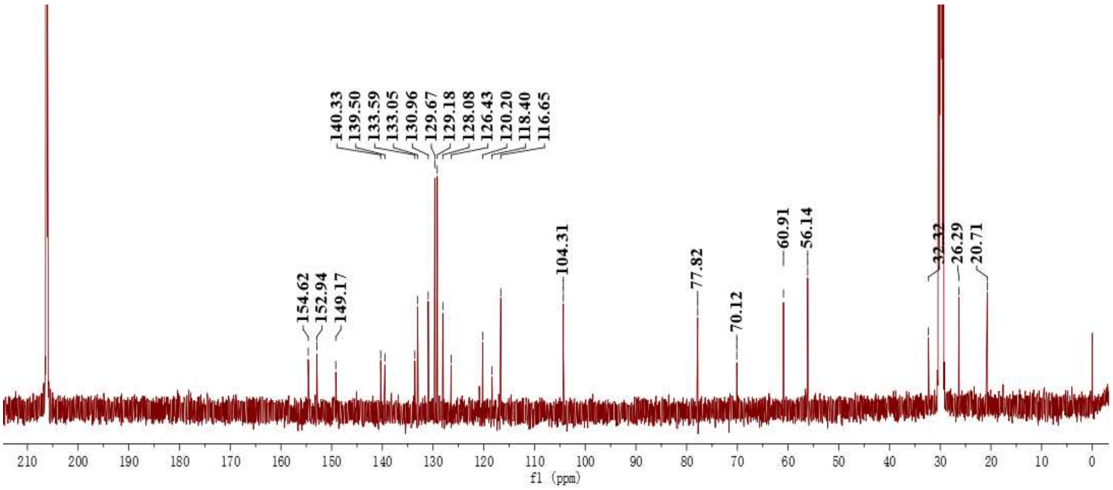

Figure S41. DEPT135 spectrum of new compound **4**

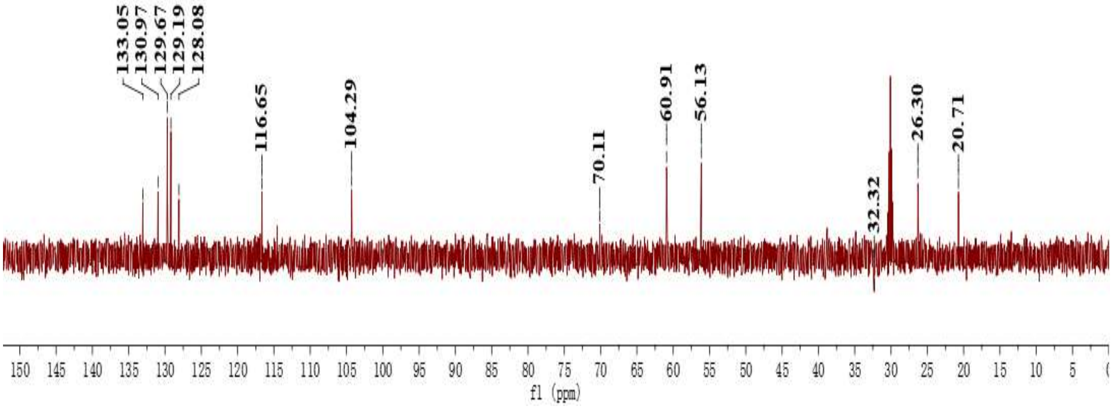

Figure S42. HSQC spectrum of new compound **4**

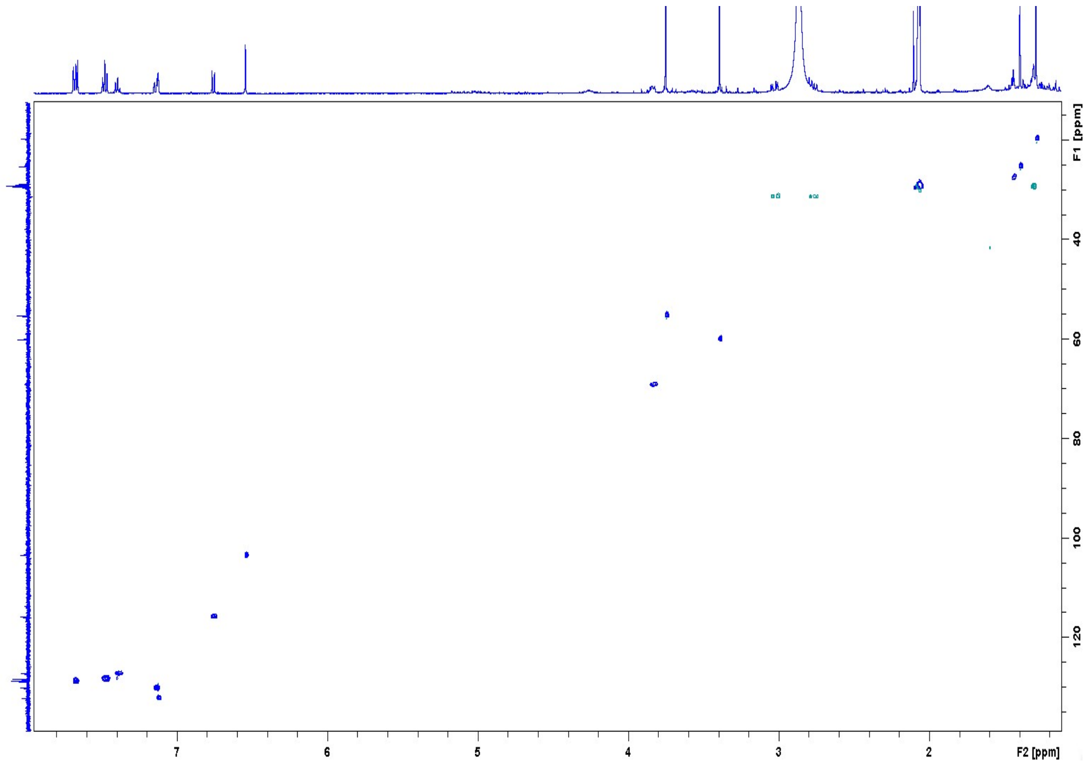

Figure S43. HMBC spectrum of new compound **4**

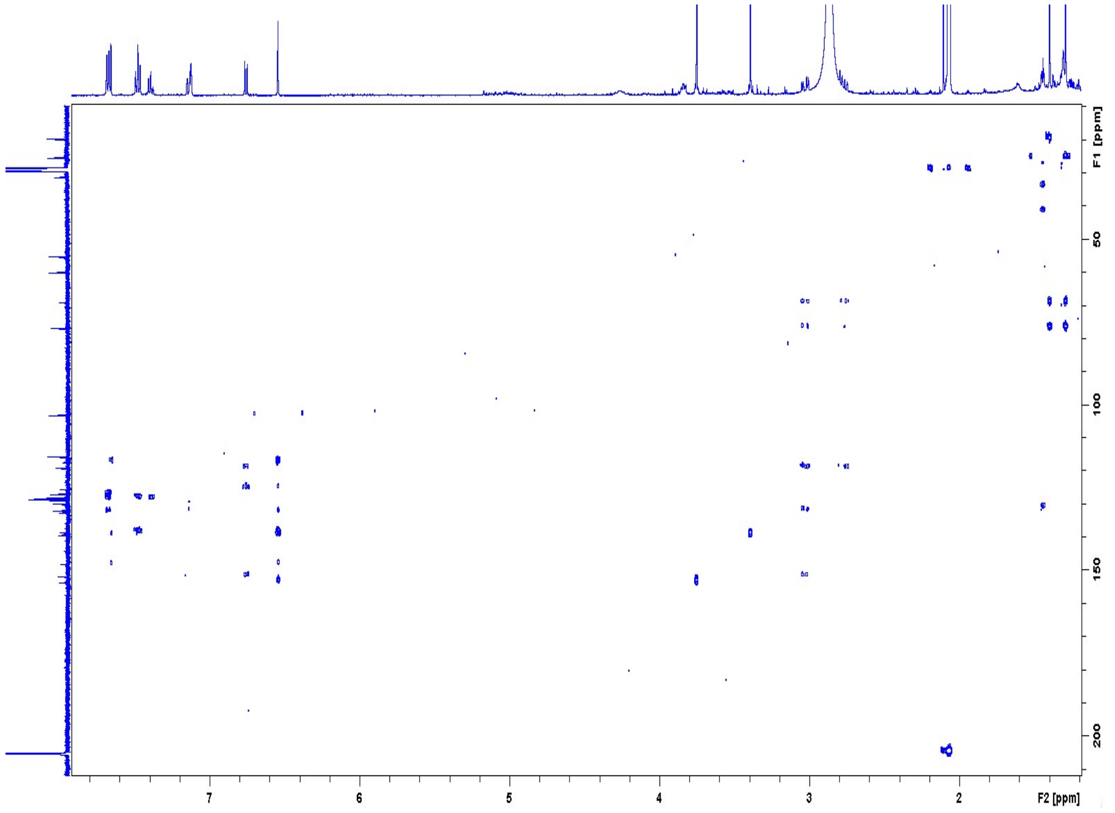

Figure S44. ^{1}H-^{1}H COSY spectrum of new compound **4**

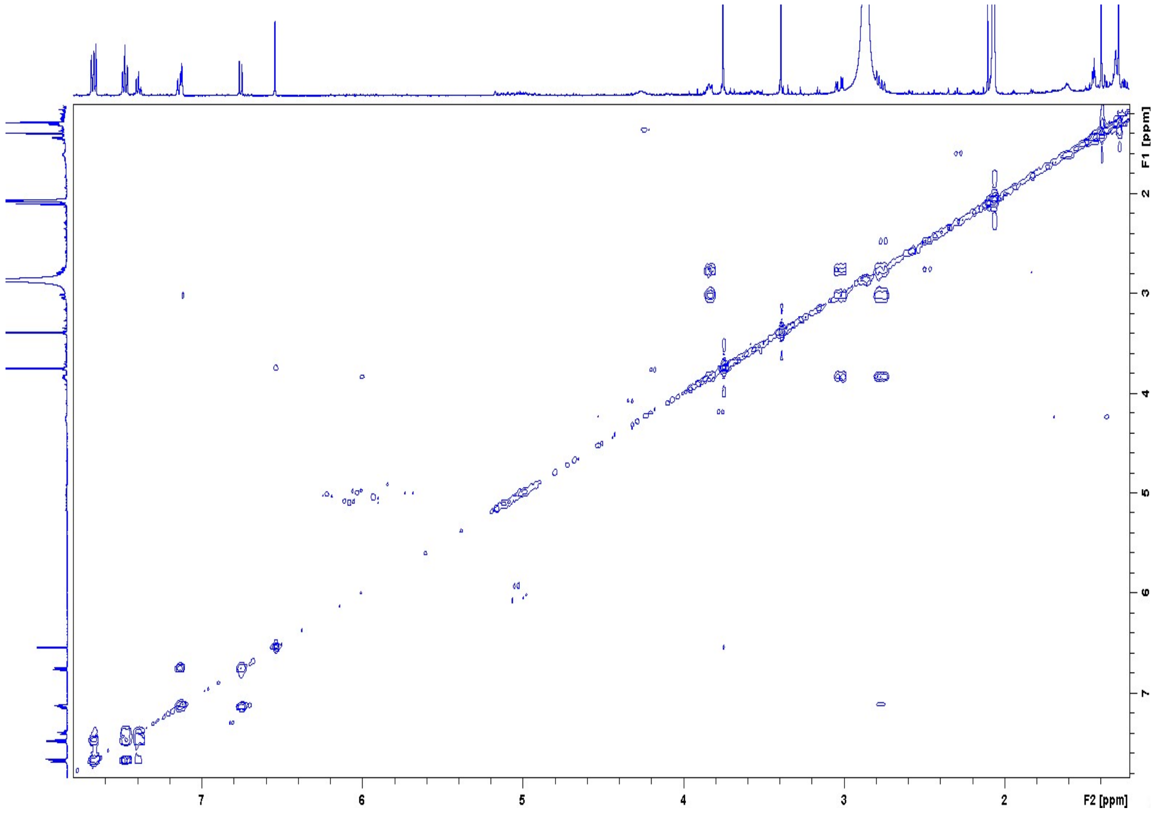

Figure S45. ROESY spectrum of new compound **4**

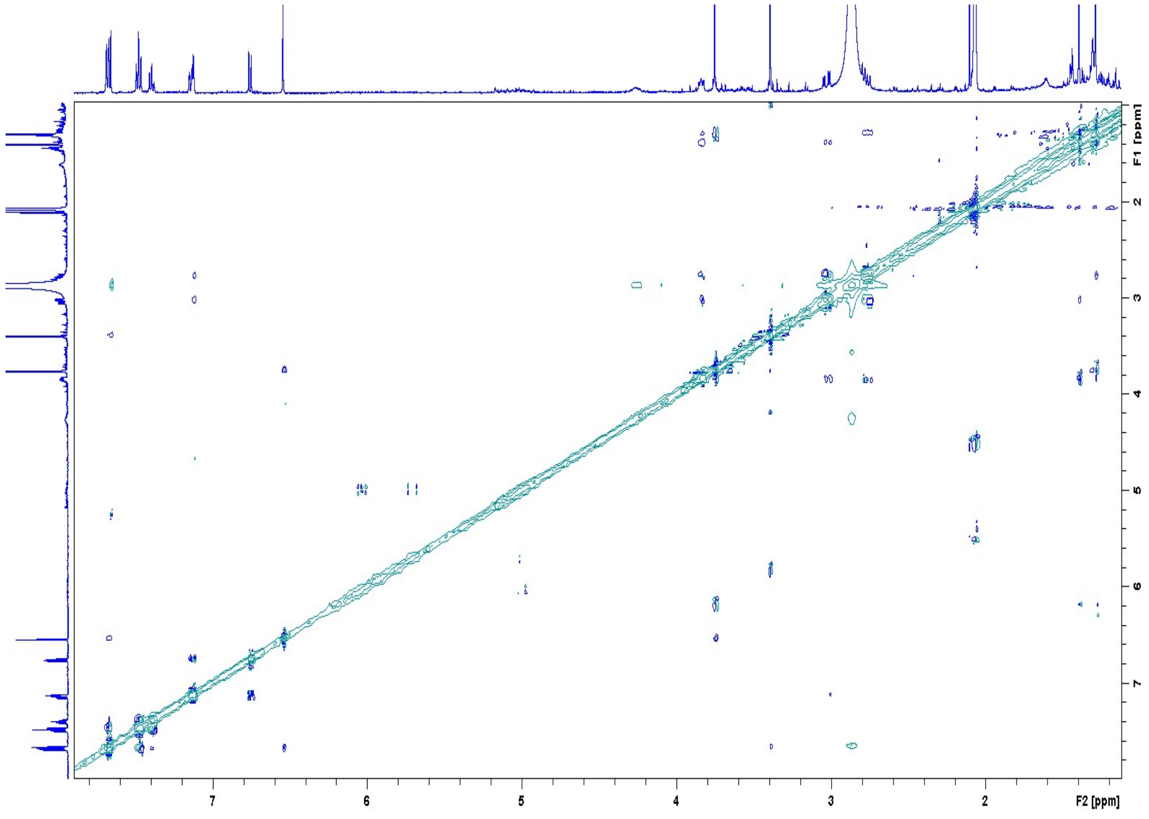

Figure S46. The HRESIMS spectrum of new compound **4**

m / z 429.1673 $[M+Na]^+$ (calcd for $C_{25}H_{26}NaO_5$, 429.1672)

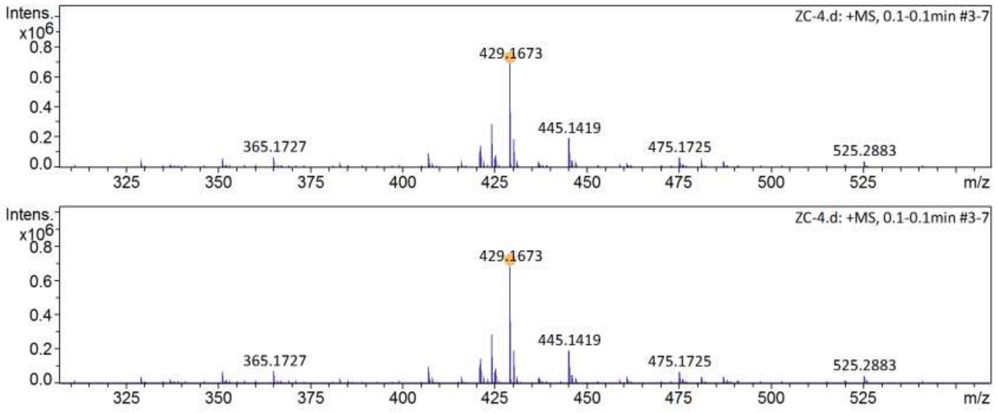

Meas. m/z	#	Ion Formula	m/z	err [ppm]	mSigma	# mSigma	Score	rdb	e⁻ Conf	N-Rule	Adduct
429.1673	1	C25H26NaO5	429.1672	-0.0	2.2	1	100.00	13.0	even	ok	M+Na

Figure S47. UV spectrum of compound **4**

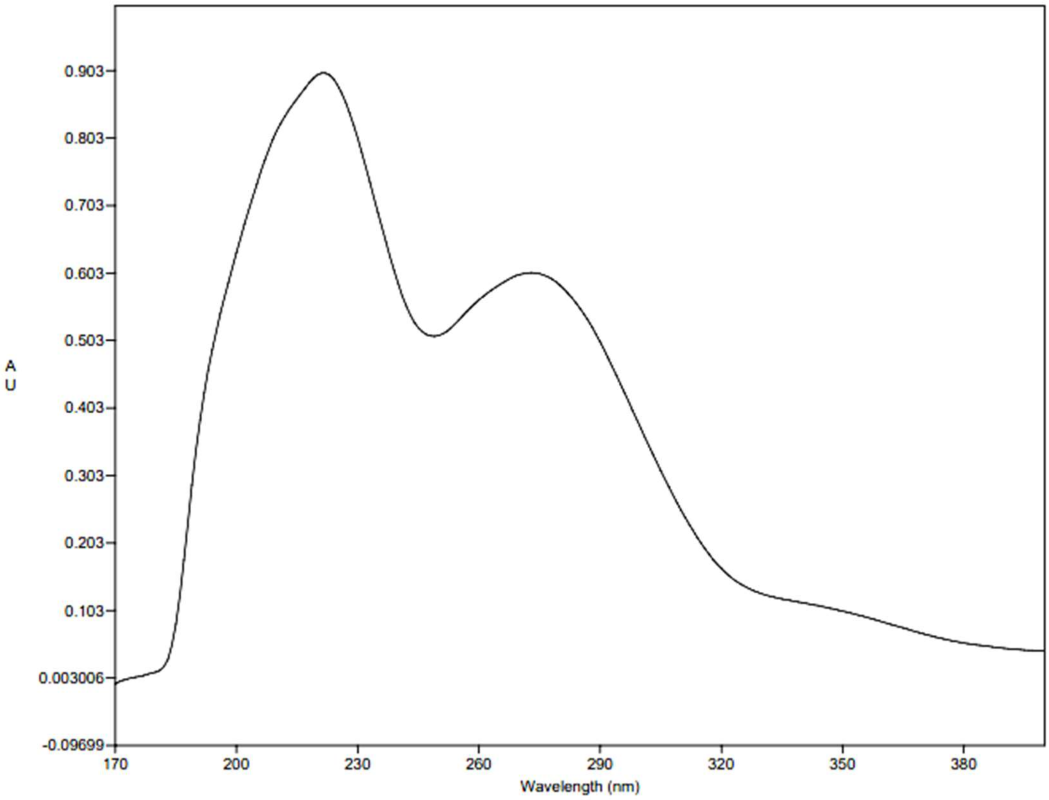

Figure S48. IR spectrum of compound **4**

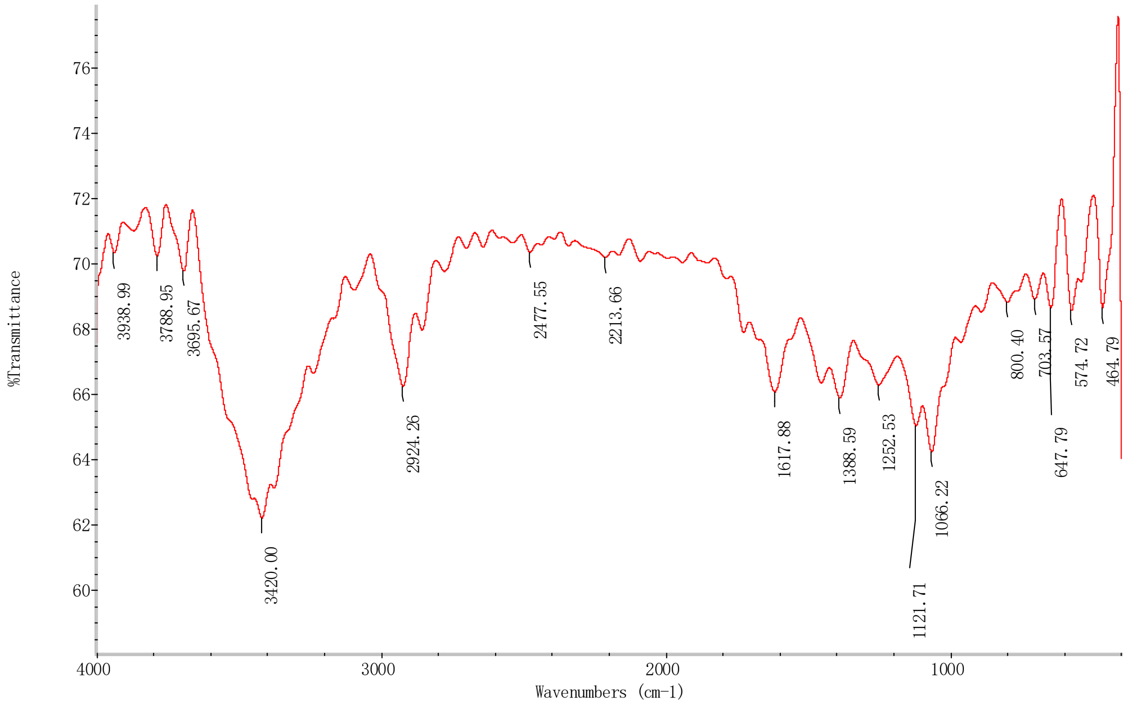

Figure S49. The ECD spectra of compounds **1-3**

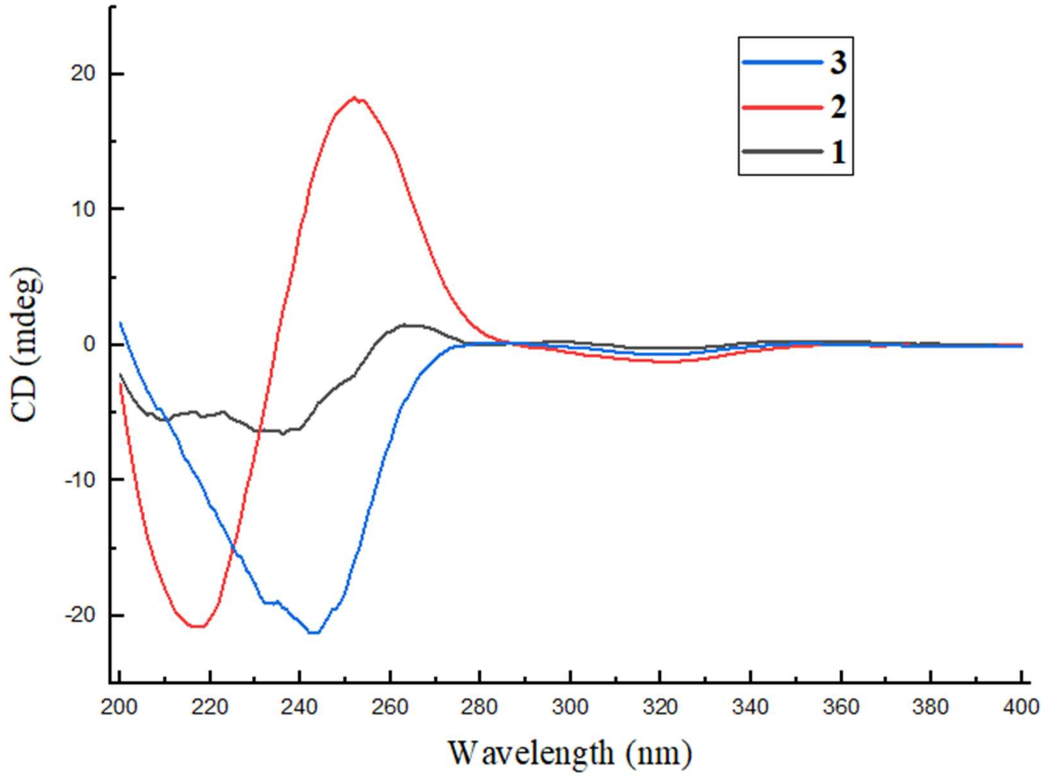

Supplement: Supplementary file 1 [file marinedrugs-22-00270-s001.zip › marinedrugs-3037324-supplementary.pdf]
